# Isotopic tracing reveals single-cell assimilation of a macroalgal polysaccharide by a few marine Flavobacteria and Gammaproteobacteria

**DOI:** 10.1038/s41396-021-00987-x

**Published:** 2021-05-05

**Authors:** François Thomas, Nolwen Le Duff, Ting-Di Wu, Aurélie Cébron, Stéphane Uroz, Pascal Riera, Cédric Leroux, Gwenn Tanguy, Erwan Legeay, Jean-Luc Guerquin-Kern

**Affiliations:** 1 Sorbonne Université, CNRS, Integrative Biology of Marine Models (LBI2M), Station Biologique de Roscoff (SBR), Roscoff, France; 2 Institut Curie, Université Paris-Saclay, Paris, France; 3 Université Paris-Saclay, INSERM US43, CNRS UMS2016, Multimodal Imaging Center, Orsay, France; 4 Université de Lorraine, CNRS, LIEC, Nancy, France; 5 Université de Lorraine, INRAE, UMR1136 « Interactions Arbres-Microorganismes », Champenoux, France; 6 Sorbonne Université, CNRS, UMR7144, Station Biologique de Roscoff (SBR), Roscoff, France; 7 CNRS, Sorbonne Université, FR2424, Metabomer, Station Biologique de Roscoff, Roscoff, France; 8 CNRS, Sorbonne Université, FR2424, Genomer, Station Biologique de Roscoff, Roscoff, France

**Keywords:** Water microbiology, Microbial ecology, Marine microbiology, Biogeochemistry, Microbial ecology

## Abstract

Algal polysaccharides constitute a diverse and abundant reservoir of organic matter for marine heterotrophic bacteria, central to the oceanic carbon cycle. We investigated the uptake of alginate, a major brown macroalgal polysaccharide, by microbial communities from kelp-dominated coastal habitats. Congruent with cell growth and rapid substrate utilization, alginate amendments induced a decrease in bacterial diversity and a marked compositional shift towards copiotrophic bacteria. We traced ^13^C derived from alginate into specific bacterial incorporators and quantified the uptake activity at the single-cell level, using halogen in situ hybridization coupled to nanoscale secondary ion mass spectrometry (HISH-SIMS) and DNA stable isotope probing (DNA-SIP). Cell-specific alginate uptake was observed for *Gammaproteobacteria* and *Flavobacteriales*, with carbon assimilation rates ranging from 0.14 to 27.50 fg C µm^−3^ h^−1^. DNA-SIP revealed that only a few initially rare *Flavobacteriaceae* and *Alteromonadales* taxa incorporated ^13^C from alginate into their biomass, accounting for most of the carbon assimilation based on bulk isotopic measurements. Functional screening of metagenomic libraries gave insights into the genes of alginolytic *Alteromonadales* active in situ. These results highlight the high degree of niche specialization in heterotrophic communities and help constraining the quantitative role of polysaccharide-degrading bacteria in coastal ecosystems.

## Introduction

By recycling a large proportion of the available organic matter, heterotrophic bacteria control the oceanic fluxes of carbon and energy [1]. The dissolved fraction is the largest reservoir of oceanic organic matter, with a global estimate of 662 Pg dissolved organic carbon (DOC) [2, 3]. Marine DOC comprises diverse low- and high-molecular-weight compounds, of which carbohydrates constitute 15–50% [4], mostly derived from micro- and macro-algae in the surface ocean. With an estimated standing stock amounting hundreds of megatons in temperate and sub-polar habitats [5, 6], brown macroalgae are major primary producers in coastal regions. Brown macroalgae of the order Laminariales, collectively known as kelps, can release up to 25% of the fixed carbon as exudates, thus contributing ~1.3 kg C m^−2^ y^−1^ to the DOC pool [7, 8]. This significantly impacts coastal ecosystems, where kelp forests locally increase DOC concentrations and sustain distinct microbial communities [9]. Previous studies measured 1–1.5 mg C L^−1^ as DOC in seawater from kelp beds [9, 10]. Kelp exudates contain simple sugars, polysaccharides, proteins, lipids, and aromatic compounds [11, 12]. In particular, the polysaccharide alginate, which comprises ca. half of kelp dry biomass [13], accounts for 5% of the released DOC [11]. Given the density and high primary productivity of kelps in coastal regions, alginate therefore constitutes an abundant resource for planktonic bacteria, available year-round but with potential seasonal variations in quantities [7]. Alginate is a linear polysaccharide consisting of β-D-mannuronic acid and α-L-guluronic acid arranged in homo- or hetero-polymeric blocks and with varying proportions of each motif depending on algal species, tissue, age, and seasons [14, 15]. Many isolated marine bacteria can degrade alginate and use it as a carbon and energy source. Alginolytic culturable representatives belong to *Proteobacteria*, *Flavobacteriales*, *Firmicutes,* and *Verrucomicrobiae* [16–19]. In particular, several polysaccharide utilization loci (PUL) dedicated to alginate assimilation have been described, which encode alginate lyases for the breakdown of the substrate into oligosaccharides, SusC/SusD homologs for binding and uptake of degradation products, and cytoplasmic enzymes processing monomers into the central metabolism [20–23]. Alginolytic PULs are widespread in marine *Flavobacteriaceae* and were transferred to *Gammaproteobacteria* [20]. Their expression is tightly controlled by PUL-encoded regulators, allowing a rapid and massive overexpression when alginate becomes available, and might be fine-tuned by yet unknown cross-regulation, catabolic repression, or substrate prioritization mechanisms [24–26]. Depending on the type and cellular localization of their alginate lyases, marine bacteria adopt different ecophysiological strategies leading to niche specialization towards polymeric alginate vs. oligomeric products [27, 28]. Despite the characterization of alginate catabolism in cultivated strains, identifying the members of natural bacterial communities that use alginate remains a challenge. Previous studies showed that alginate amendments induce shifts in seawater community composition [29–32] and provided insights into the global community response and alginolytic potential. However, these approaches cannot directly link alginate utilization to individual bacterial taxa nor estimate substrate assimilation at the single-cell level, hindering our comprehension of alginate fate in marine ecosystems. To date, few studies focused on the direct detection and quantification of the utilization of a given substrate by individual bacteria in natural samples, using e.g., microautoradiography, Raman spectroscopy, fluorescently-labeled substrates, or stable isotope probing (SIP) [33, 34]. To our knowledge, SIP has seldom been applied using complex marine organic matter [35–39], and never to macroalgae-derived polysaccharides, despite their importance in the carbon cycle. Here, we combined DNA-SIP with halogen in situ hybridization coupled to chemical imaging via nanoscale secondary ion mass spectrometry (HISH-SIMS) to trace ^13^C-labeled alginate into specific incorporators within a coastal seawater community and quantify the uptake activity at the single-cell level. We show that most of the alginate is used by a few initially rare *Flavobacteriaceae* and *Gammaproteobacteria* taxa and provide the first single-cell estimates of alginate incorporation rates.

## Methods

### Alginate production

Natural (^12^C-natural) and ^13^C-enriched alginates were prepared as described previously [40]. Briefly, the kelp *Laminaria digitata* was cultivated with either natural (98.9 ^12^C%) or ^13^C-labeled (99 ^13^C%) NaHCO_3_. Alginate was chemically extracted as detailed in Supplementary Methods. The average molecular weight of alginate estimated using multi-angle laser light scattering (MALLS) was Mw = 1.7 10^5^ Da. Alginates were assayed for residual proteins and DNA using the Qubit Protein Assay kit and dsDNA HS assay kit (ThermoFisher Scientific), respectively. ^12^C-natural alginate contained 13.7 mg protein and 284 µg DNA g^−1^ (i.e., 98.6% purity). ^13^C-enriched alginate contained 10.1 mg protein and 288 µg DNA g^−1^ (i.e., 99.0% purity). The absence of inhibitors was confirmed by checking the growth of the alginolytic strain *Zobellia galactanivorans* Dsij^T^ in minimum medium supplemented with ^12^C-natural or ^13^C-enriched alginate (1 g l^−1^). Alginate isotopic ratio was measured by elemental analysis coupled to isotope ratio mass spectrometry (EA-IRMS) as described previously [40]. ^12^C-natural and ^13^C-enriched alginate had 1.1337 ± 0.0004 and 3.6279 ± 0.0009 ^13^C atom percent, respectively (mean ± s.d. *n* = 3 technical replicates). Before microcosm amendment, alginates were solubilized (5 g l^−1^) in Tris-HCl 50 mM pH 8.0 and autoclaved. The average molecular weight of autoclaved alginate determined by MALLS analysis was Mw = 1.4 10^5^ Da with a polydispersity index of 1.364, corresponding to an average degree of polymerization of ca. 800 monomers.

### Seawater microcosms

Natural seawater was collected at 12.30 pm on January 21 2019 from a kelp-dominated tidal pool at Le Bloscon, Roscoff, France (48°43'33.18“N, 3°58'7.58“W). Average surface water temperature, salinity, and pH are 10–12 °C, 35.0–35.2 g kg^−1^,and 7.75–7.80 in January in this area (values from long-term monitoring SOMLIT Estacade Station, 1.2 km from sampling site). Autoclaved plastic carboys were rinsed three times with water from the site before sampling. Seawater from different carboys was pooled and homogenized in a sterile container. Three aliquots (950 ml) were filtered for DNA extraction at T0. The remaining seawater was distributed in 1-liter aliquots into sterile 5-liter flasks with 0.1% marine ammonium mineral salts (DSMZ #1313). Microcosms were amended with either ^12^C-natural alginate (*n* = 3) or ^13^C-enriched alginate (*n* = 3) at 20 mg l^−1^, corresponding to ca. 8 mg C l^−1^. Unamended controls (*n* = 3) were prepared by adding the same volume of Tris-HCl 50 mM pH 8.0 without alginate. All flasks were incubated at 15 °C, 130 rpm in the dark. The set-up was complete within 3 h after sampling. Aliquots were sampled from all microcosms at *T* = 0, 18.5, 24.5, 42.5, and 47 h for flow cytometry and uronic acid measurements, and only at *T* = 47 h for bulk isotope, CARD-FISH and HISH-SIMS analyses, and DNA extraction.

### Flow cytometry

Aliquots (250 µl) were fixed with 1.25 µl glutaraldehyde 50% and diluted with an appropriate volume of 1X TE buffer containing SybrGreen (Life Technologies, 2 µl for 15 ml TE buffer). Cells were counted in technical triplicates on BD Accuri C6 Plus Flow Cytometer.

### Uronic acid measurements

Aliquots (1 ml) were centrifuged 10 min at 7000 rpm. To follow alginate concentrations, uronic acids were quantified in supernatants using the meta-hydroxy-di-phenyl (MHDP) method [41]. Samples (200 µl) were mixed with 20 µl of 4 M sulfamic acid and 1.2 ml of 75 mM sodium tetraborate. After incubation (20 min, 80 °C), 40 µl of MHDP 0.15% were added. OD_525_ was measured after 10 min on a spectrophotometer and compared to a standard curve of glucuronic acid from 2.5 to 100 µg ml^−1^.

### CARD-FISH and HISH-SIMS analyses

Aliquots (1.8 ml) were preserved with paraformaldehyde (1%, 1 h at room temperature) and stored in PBS/ethanol 1:1 (v/v) at −20 °C. Fixed cells (500 µl aliquots) were filtered under moderate vacuum on 0.2 µm polycarbonate membranes (Isopore) previously sputtered with a 80/20 Au/Pd alloy, and washed twice with 5 ml PBS. Filters were air-dried and stored at −20 °C until analysis. Hybridization procedures were performed as described previously [42]. Filters were embedded in 0.1% low-melting point agarose. Cells were permeabilized with 10 mg ml^−1^ lysozyme in TE buffer (30 min, 37 °C). Endogenous peroxidases were inactivated in 3% H_2_O_2_ (10 min, room temperature). Hybridizations were performed at 46 °C for 3 h in 300 µl buffer (35% formamide) containing 28 nM of the following HRP-labeled probes: GAM42a (with competitor BET42a) targeting most *Gammaproteobacteria* [43], CF319a targeting most *Flavobacteriales* and other members of the CFB group [44] and NON338 used as a negative control [45]. Signal amplification was conducted at 46 °C for 45 min, using fluorine-containing tyramides synthesized from OregonGreen 488-X succinimidyl ester (Molecular Probes) as described previously [46]. Portions of hybridized filters were stained with DAPI (1 µg ml^−1^) before bacterial counts on an Olympus BX60 microscope with epifluorescence irradiation.

HISH-SIMS analysis was performed using a NanoSIMS-50 Ion microprobe (CAMECA, Gennevilliers, France) in scanning mode [47, 48] (details in Supplementary Methods). After Cs^+^ pre-implantation, five secondary ions were monitored: ^12^C^−^, ^19^F^−^, ^12^C^14^N^−^, ^13^C^14^N^−^, and ^32^S^−^. Images of ^32^S^−^, [^19^F^−^]/[^32^S^−^] and ^13^C atom fraction were processed using ImageJ [49]. The ^13^C atom fraction map was established from ^12^C^14^N^−^ and ^13^C^14^N^−^ images based on pixel-by-pixel calculation as follows:

^13^C At% = [^13^C^14^N^−^]/([^12^C^14^N^−^] + [^13^C^14^N^−^]) x 100%

Single-cell carbon assimilation rates were inferred from NanoSIMS data, following calculations developed in [50] and detailed in Supplementary Methods. Briefly, the fraction of carbon assimilated (*K*_*A*_) during incubation in microcosms containing ^13^C-enriched alginate was calculated for each selected cell, considering a carbon dilution of 29.38% due to the CARD-FISH treatment [51]. Volume-specific carbon assimilation rates were inferred from *K*_*A*_ values based on the measured biovolume for each cell, the incubation time, and the partial density of carbon in bacterial cells.

### Bulk isotopic analysis

Aliquots (1 ml) were filtered onto precombusted GF filters (0.45 µm pore size), which were frozen at −20 °C until EA-IRMS analysis. Carbon isotopic ratios (R = ^13^C/^12^C) were determined on filters folded into tin capsules, using a CHN analyzer (ThermoFinnigan 1112 Series) interfaced with a mass spectrometer (ThermoFinnigan MAT Delta Plus) via a Conflow III open split interface. Abundances were calculated in relation to Vienna Pee Dee Belemnite-limestone (V-PDB), using in-house casein standards calibrated against IAEA-600 and IAEA-CH-6 international standards.

Bulk carbon assimilation was calculated as atom percent excess (APE) as follows:

$$APE = 100 \times \left( {\frac{{R_f}}{{R_f + 1}} - \frac{{R_i}}{{R_i + 1}}} \right)$$

where *R*_*f*_ and *R*_*i*_ are the ^13^C isotope ratio at the final and initial sampling time, respectively.

Bulk carbon incorporation rates (*F*_bulk_) were calculated as follows:

$$F_{bulk} = \frac{{K_A \times \rho _C \times A}}{t}$$

where *K*_*A*_ is the fraction of assimilated carbon (calculated as described in Supplementary Methods), *ρ*_*C*_ is the amount of carbon per cell, *A* is the final cell abundance estimated through flow cytometry and *t* is the incubation time. We considered a range of *ρ*_*C*_ = 10–280 fg C cell^−1^ [50].

### DNA extraction

Seawater (950 ml) was filtered on 0.22 µm Sterivex-GP polyethersulfone filters (Merck) using a peristaltic pump under moderate flow with a 3 µm pre-filter. DNA was extracted using the NucleoSpin PlantII kit (Macherey–Nagel) as in Ramond et al. [52], eluted in 100 µl and quantified using a Qubit fluorometer.

### Isopycnic centrifugation and fractionation

DNA from ^12^C-natural and ^13^C-enriched alginate-amended microcosms was fractionated on CsCl density gradients as described previously [53]. DNA (5 µg) was mixed with gradient buffer (0.1 M Tris-HCl, 0.1 M KCl, 1 mM EDTA) and CsCl solution to a final buoyant density (BD) of 1.725 g ml^−1^. Ultracentrifugation was performed in a vertical rotor (VTi 65.2, Beckman), at 15 °C, 176,985 g for 64 h. Sixteen fractions of 340 µl were collected from each tube. The refractive index (RI) of each fraction was measured with a refractometer (VWR) and corrected to account for the gradient buffer RI using the equation $RI_{corrected} = RI_{observed} - (RI_{buffer} - 1.3333)$ [54]. BD was calculated from corrected RI using the equation $BD = a \times RI - b$ [55] where *a* and *b* are coefficients for CsCl at 20 °C (*a* = 10.9276; *b* = 13.593) [56]. The average difference in BD between successive fractions was 0.0028. DNA was recovered by overnight precipitation with 20 µg glycogen (MP Biomedicals) and 700 µl of polyethylene glycol solution (30% PEG 6000, 1.6 M NaCl) followed by centrifugation for 45 min at 13,000 g. DNA was rinsed with 70% ethanol, air-dried, and resuspended in 30 µl of molecular-biology grade water.

### 16S rRNA gene metabarcoding

Library preparation and sequencing were performed as described previously [57] and detailed in Supplementary Methods. A 464-bp fragment of 16S rRNA genes was amplified from non-fractionated total DNA samples [T0 (*n* = 3), ^12^C-natural alginate (T47-ALG12, *n* = 3), ^13^C-enriched alginate (T47-ALG13, *n* = 3) and unamended controls (T47-CTRL, *n* = 3)] and gradient fractions (*n* = 14 fractions per gradient) using primers S-D-Bact-0341-b-S-17 and S-D-Bact-0785-a-A-21 [58]. The library was prepared using the Nextera XT DNA library prep kit (Illumina). MiSeq sequencing (2 × 300 cycles, Illumina) yielded a total of 7,250,023 paired-end sequences from 96 samples (accessible at NCBI under BioProject accession PRJNA686971). Quality-filtered reads were processed using default parameters of DADA2 [59] implemented in QIIME 2 v2018.8 [60] and clustered de novo in operational taxonomic units (OTUs) at 97% using vsearch [61]. Taxonomy was assigned using SILVA ssu132 Ref NR99. OTUs representing <0.001% of total sequences or affiliated to chloroplasts, mitochondria, or archaea were discarded (2.6% of input sequences). The final dataset comprised 1074 OTUs and 2,909,992 sequences, ranging from 12,948 to 174,305 sequences per sample. Data were further analyzed in phyloseq [62]. Alpha-diversity indices for non-fractionated samples were calculated on the non-transformed dataset. Beta-diversity analyses were performed on Hellinger-transformed datasets using PCoA on a weighted Unifrac distance matrix. The effect of alginate amendments on total community structure was tested using PERMANOVA with 999 permutations. Differential abundance analysis between unamended controls (*n* = 3) and alginate-amended microcosms (*n* = 6) was performed using DESeq2 [63] based on the negative binomial distribution with Wald test and parametric fitting. Differences were considered significant when Benjamini–Hochberg corrected *p* were <0.05 and log_2_ fold-change >1.

### DNA-SIP

For DNA-SIP analysis, we retained OTUs that were present at least once in each of the three gradients from ^13^C-enriched samples. This filtered dataset comprised 183 OTUs and 2,173,323 sequences, i.e., 98% of the data obtained from gradient fractions. Analysis was performed with HTSSIP v1.4.1 [64] using multiple-windows high-resolution SIP (MW-HR-SIP) [54, 65], with a sparsity cutoff of 0.25, a log_2_ fold-change null threshold of 0.25 and a significance threshold *α* = 0.05. Briefly, MW-HR-SIP identifies incorporators by utilizing DESeq2 to detect OTUs that have a higher relative abundance in multiple overlapping “heavy” BD windows of ^13^C-enriched vs. ^12^C-natural alginate gradients. Four heavy BD windows were tested (1.715–1.730, 1.720–1.735, 1.725–1.740, 1.730–1.745). Representative sequences of each ^13^C-incorporating OTU were analyzed using blastn on the NCBI 16S rRNA database.

### Metagenomic fosmid library

A metagenomic fosmid library was prepared from 13.5 µg unfractionated DNA from alginate-amended microcosms following the CopyControl Fosmid Library production kit protocol (Epicentre). Briefly, end-repaired DNA was cloned into the pCC2FOS vector and transfected in *Escherichia coli* EPI300-T1^R^. The clone library was recovered on LB-agar containing chloramphenicol (12 µg ml^−1^) overnight at 37 °C, and stored at −80 °C in 96-well microplates. Clones were screened on LB-agarose containing chloramphenicol (12 µg ml^−1^), arabinose (0.2 g l^−1^), and sodium alginate (7 g l^−1^). After 4 days at 37 °C, plates were flooded for 10–30 min with 10% cetylpyridinium chloride to detect alginolytic activity as clearing zones against an opaque background. Recombinant alginate lyase AlyA1 from *Zobellia galactanivorans* Dsij^T^ [66] was used as positive control. Sequencing libraries were prepared from positive fosmid DNA using the Nextera XT DNA kit (Illumina) and sequenced using MiSeq v3 PE300 (Illumina). Reads were assembled using SPAdes v3.11 [67], resulting in one contig of 42,237 bp, including 34,093 bp of metagenomic insert and 8144 bp of pCC2FOS for fosmid F10, and one contig of 51,052 bp, including 42,696 bp of metagenomic insert and 8356 bp of pCC2FOS for fosmid F25. The F10 and F25 metagenomic insert sequences were deposited under Genbank accessions MW442085 and MW442086, respectively. ORFs were predicted and annotated using RAST [68] and prokka [69]. Protein sequences were analyzed using blastp on the nr database. Predicted alginate lyases were annotated using dbcan [70], CUPP [71], SignalP 5.0 [72] and Pfam [73].

## Results

### Dynamics of alginate utilization and effect on bacterial community

**Fig. 1:**
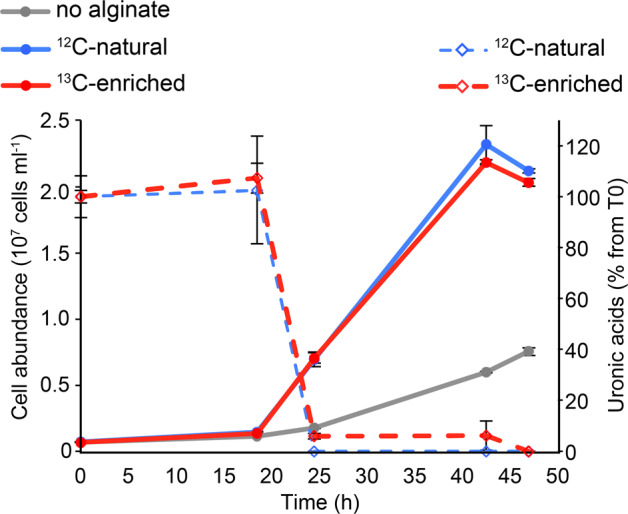
Monitoring of growth and substrate utilization in microcosms amended with either ^12^C-natural (blue) or ^13^C-enriched alginate (red), or in unamended controls (grey). Cell abundance was measured by flow cytometry (circles and plain lines, left axis). Alginate consumption was measured as the percentage of added uronic acids that remained in microcosms over time (diamonds and broken lines, right axis). Values are mean ± s.e.m. (*n* = 3).

Cell abundance increased from 6.82 ± 0.06 10^5^ to 2.25 ± 0.07 10^7^ cell ml^-1^ in both ^12^C or ^13^C- alginate-amended treatments (Fig. 1). Final cell abundance was 3-fold lower in unamended controls compared to alginate-amended microcosms. Most of the added alginate (95-100%) was consumed between 18 and 25 h of incubation.

Two-days incubation strongly decreased the richness (Chao1 index) and diversity (Shannon and Simpson index) of the seawater bacterial community compared to its initial state (Figure S1A). The community was more diverse in alginate-amended microcosms compared to unamended controls (Welch’s *t*-test, *p* = 0.002 and *p* = 0.021 for Shannon and Simpson, respectively), although one of the microcosms amended with ^13^C-enriched alginate was an outlier with low diversity. Furthermore, the OTU-level community structure of alginate-amended microcosms differed strongly from that of the initial community and unamended microcosms (Figure S1B, PERMANOVA, *F* = 14.8, *p* = 0.002). There was no significant effect of the alginate type (^12^C-natural vs. ^13^C-enriched) on the final community structure (PERMANOVA, *F* = 0.64, *p* = 1).

**Fig. 2:**
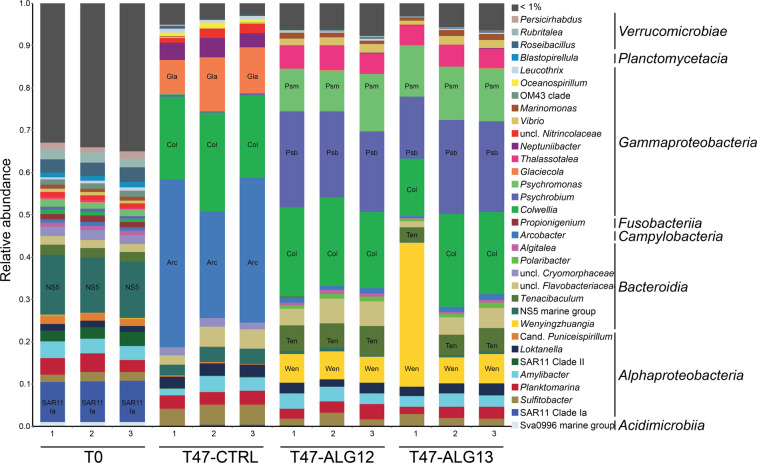
Taxonomic composition of bacterial communities based on 16S rRNA gene analysis of non-fractionated DNA, shown at the genus and class levels. Individual plots are shown for triplicate incubations in each condition. Genera individually representing less than 1% relative sequence abundance have been collapsed in the “<1%” category. Abbreviations are shown on the bars for abundant groups discussed in the text. Arc *Arcobacter*; Col *Colwellia*; Gla *Glaciecola*; Wen *Wenyingzhuangia*; Ten *Tenacibaculum*; Psb *Psychrobium*; Psm *Psychromonas*.

A total of 389 genera were detected in the seawater bacterial community (Fig. 2). The initial community was dominated by the *Flavobacteriaceae* NS5 marine group (relative sequence abundance 13.5 ± 0.4%, mean ± s.e.m., *n* = 3) and the alphaproteobacterial SAR11 Clade Ia (9.6 ± 0.1%), *Planktomarina* (3.7 ± 0.5%) and *Amylibacter* (3.6 ± 0.2%). Incubation for 47 h with or without alginate amendment induced drastic shifts in taxonomic composition. The unamended controls were dominated by the *Epsilonproteobacteria* genus *Arcobacter* (33.1 ± 4.2%), followed by *Colwellia* (20.7 ± 1.3%) and *Glaciecola* (10.6 ± 1.4%) within the order *Alteromonadales*. Such a short-term “bottle effect” has previously been observed for seawater microcosms [74] and might be due to partial oxygen depletion, modified nutrient availability, or accumulation of metabolites in sealed containers compared to open environments. Overall, similar taxonomic composition was observed in all the alginate-amended microcosms irrespective of isotope enrichment. The most prevalent genera were *Psychrobium* (20.1 ± 1.2%, *n* = 6), *Colwellia* (19.2 ± 1.2%) and *Psychromonas* (11.6 ± 0.6%) within *Alteromonadales* (*Gammaproteobacteria*), as well as *Wenyingzhuangia* (11.1 ± 4.6%) and *Tenacibaculum* (5.4 ± 0.5%) within *Flavobacteriaceae* (*Bacteroidia*). One replicate microcosm amended with ^13^C-enriched alginate (T47-ALG13-1) partly differed from the other alginate-amended microcosms due to a strong enrichment in the genus *Wenyingzhuangia* that accounted for 34% of the sequences. The prevalence of *Gammaproteobacteria* and *Flavobacteriales* cells in microcosms amended with ^13^C-enriched alginate was confirmed by CARD-FISH, with final proportions reaching 45 ± 11 and 25 ± 9%, respectively (Table S1).

A total of 64 OTUs belonging to 28 genera were found significantly more abundant in alginate-amended microcosms (*n* = 6) compared to unamended controls (*n* = 3), while 15 OTUs were significantly more abundant without amendment (Figure S2). Most of the OTUs significantly enriched in alginate-amended microcosms belonged to *Gammaproteobacteria* (34/64, including 24 *Alteromonadales*) and *Bacteroidia* (28/64, including 27 *Flavobacteriales*). The most alginate-responsive OTUs were affiliated to *Psychrobium*, *Wenyingzhuangia,* and *Colwellia*.

### Signals of ^13^C-assimilation at the community and single-cell levels

**Fig. 3:**
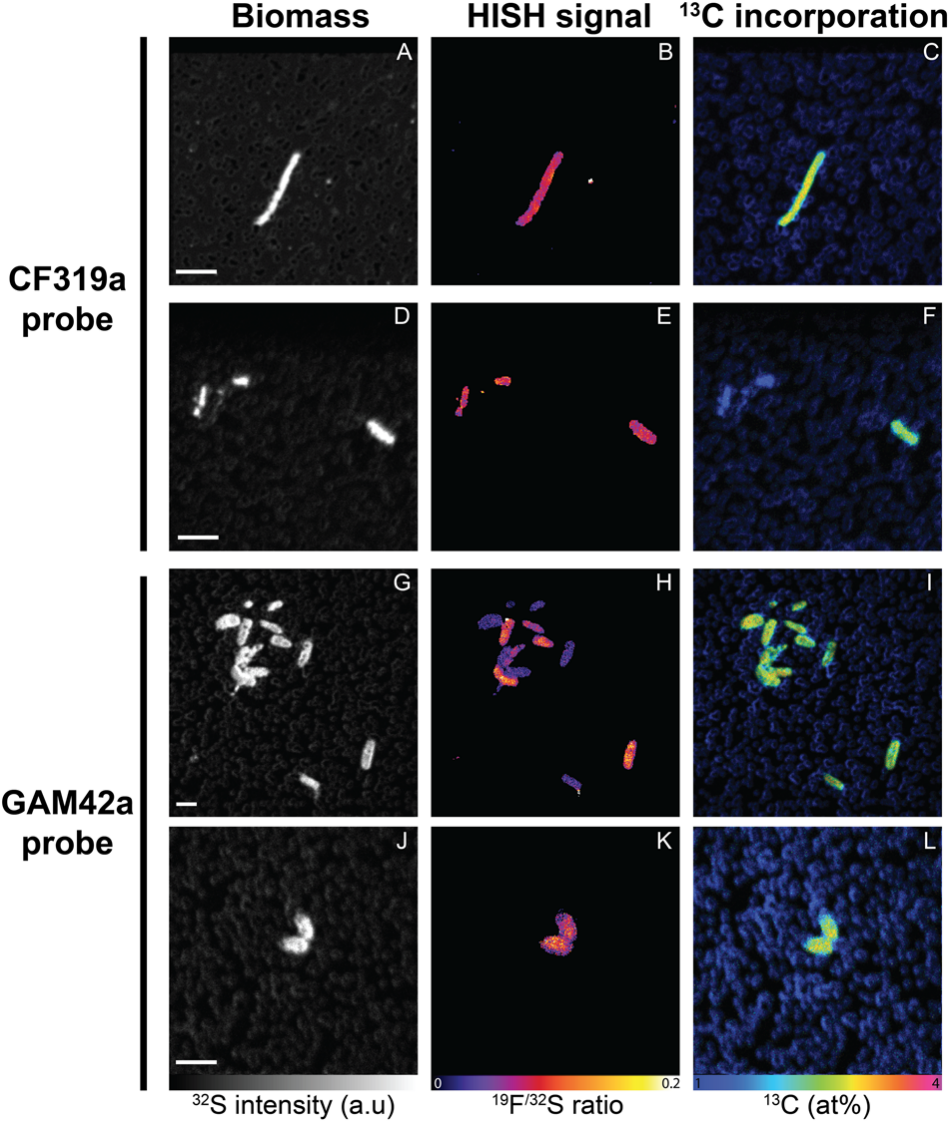
NanoSIMS analysis of cells from microcosms amended with ^13^C-enriched alginate. Cells were hybridized with CF319a probe targeting *Flavobacteriales* (**A**–**F**) or GAM42a probe targeting *Gammaproteobacteria* (**G**–**L**), using fluorine-containing tyramides. Rows show parallel acquisitions of the same region. Columns display secondary ion images of ^32^S as total biomass indicator (**A**, **D**, **G,** and **J**; a.u: arbitrary intensity unit), the ratio ^19^F/^32^S as a marker for cell identity (**B**, **E**, **H,** and **K**) and the ^13^C atom fraction inferred from secondary ions (^13^C^14^N, ^12^C^14^N) as indicator of ^13^C incorporation from alginate (**C**, **F**, **I,** and **L**), in HSI (Hue-Saturation-Intensity) color scale. Scale bars: 2 µm.

**Fig. 4:**
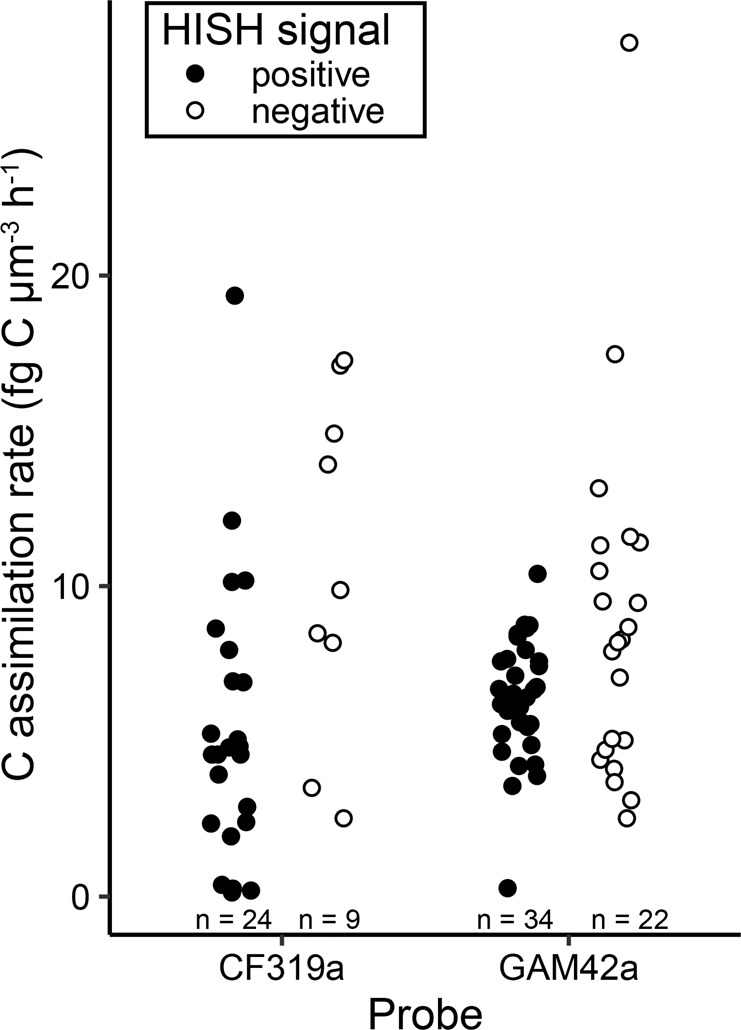
Volume-specific carbon assimilation rates calculated for ^13^C-enriched cells from alginate-amended microcosms. Results are shown for cells with a positive (black) or negative (white) hybridization with the CF319a or GAM42a probes. The number of analyzed single cells is given for each condition.

We analyzed carbon incorporation in microcosms amended with ^13^C-enriched alginate, by measuring bulk ^13^C uptake with EA-IRMS and cell-specific uptake with HISH-SIMS. Bulk samples showed a significant ^13^C At% excess of 0.34 ± 0.05. Using lower and upper estimates of 10–280 fg C cell^−1^ [50], extrapolated bulk rates of carbon incorporation from alginate ranged from 16 ± 3 to 445 ± 81 µg C L^−1^ d^−1^. Based on the strong enrichment in *Flavobacteriales* and *Gammaproteobacteria* following alginate amendment, HISH-SIMS analysis was focused on these two groups using probes CF319a and GAM42a, respectively. Cells from both groups showed significant ^13^C enrichment (Fig. 3, Table S2, Figure S3). ^13^C-enriched GAM42a-positive cells were all short and thick rods (34 analyzed cells, *L* = 1.56 ± 0.09 µm, *W* = 0.96 ± 0.05 µm). Two morphologies were detected for ^13^C-enriched CF319a-positive cells: (i) thin rods with length <1.2 µm (13/24 analyzed cells, *L* = 0.79 ± 0.05 µm, *W* = 0.34 ± 0.03 µm) and (ii) longer rods reaching up to 6.5 µm (11/24 analyzed cells, *L* = 2.42 ± 0.45 µm, *W* = 0.65 ± 0.11 µm, see example in Fig. 3A-C). ^13^C-enriched cells showed atom percent enrichment from 0.03 to 2.33 at %. Volume-specific carbon assimilation rates ranged from 0.14 to 27.50 fg C µm^−3^ h^−1^ (Fig. 4). The average carbon assimilation rate did not differ significantly between *Flavobacteriales* and *Gammaproteobacteria* (5.43 ± 0.91 and 6.34 ± 0.33 fg C µm^−3^ h^−1^, Welch’s *t*-test, *t* = −0.94, *p* = 0.35), but values were significantly more dispersed for *Flavobacteriales* (*F* test of variance, *F* = 5.43, *p* < 0.001).

### DNA-SIP identification of alginate incorporators

**Fig. 5:**
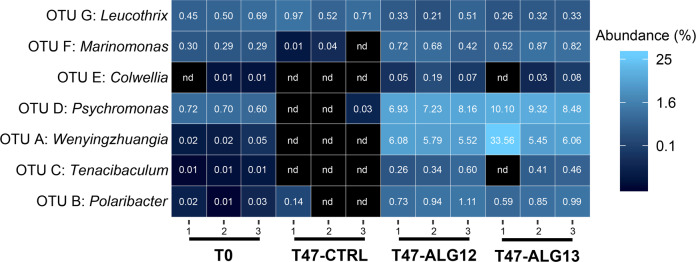
Heatmap of the relative sequence abundance of ^13^C-incorporating OTUs in the total communities based on 16S rRNA gene metabarcoding of non-fractionated DNA. Triplicates of the different conditions are shown in separate columns. nd not detected.

To specifically identify cells deriving their carbon from alginate, we performed a DNA-SIP analysis of samples collected after 47 h of incubation. Seven OTUs were detected as incorporators of ^13^C from alginate, all affiliated with *Flavobacteriaceae* and *Gammaproteobacteria* (Table 1). The strongest signals (difference in relative abundance in heavy gradient fractions of ^13^C-enriched samples compared to corresponding fractions of ^12^C-natural samples) were found for OTU A (log_2_-fold change = 2.30) affiliated to *Wenyingzhuangia* and OTU E (log_2_-fold change = 2.31) affiliated to *Colwellia* (Figure S4). We further investigated the variation of relative abundance for these seven OTUs in total bacterial communities (Fig. 5). They all were initially at low relative sequence abundance in the sampled seawater (range 0–0.72%). For all but one ^13^C-incorporating OTU, the relative abundance increased in alginate-amended microcosms while it decreased in unamended controls (range 0–0.14%). The only exception was OTU G affiliated to *Leucothrix*, whose abundance stayed stable in all conditions tested (0.21–0.97%). After 47 h with alginate, two ^13^C-incorporating OTUs became dominant in the total bacterial community, namely OTU A (*Wenyingzhuangia*, final relative abundance 10.4 ± 4.6%, *n* = 6) and OTU D (*Psychromonas*, 8.4 ± 0.5%). Combining these results with final cell counts and NanoSIMS-derived cell-specific carbon assimilation rates, we estimated the carbon assimilation attributed to the ^13^C-incorporating OTUs identified by HTS-DNA-SIP. In total, the estimate of carbon assimilation by the seven ^13^C-incorporating OTUs was 438 ± 62 µg C L^−1^ d^−1^, at the upper end of the estimate obtained from bulk analysis. Collectively, the three *Flavobacteriaceae* OTUs A, B, and C accounted on average for 146 ± 55 µg C L^−1^ d^−1^. This estimate of carbon assimilation was significantly higher (Welch *t*-test, *t* = −2.63, *p* = 0.043) for the four *Gammaproteobacteria* OTUs D, E, F, and G, which collectively accounted for 292 ± 12 µg C L^−1^ d^−1^.

**Table 1:** List of OTUs detected as ^13^C incorporators using MW-HR-SIP.

OUT code	Family	Genus	BD window^a^	log_2_FC^b^	Best blast hit		
					Strain	%ID	Isolation source
A	*Flavobacteriaceae*	*Wenyingzhuangia*	1.730-1.745	2.30	*W. fucanilytica* strain CZ1127	98.1	shallow coastal seawater, China
B	*Flavobacteriaceae*	*Polaribacter*	1.720-1.735	0.62	*Po. lacunae* strain HMF2268	100	lagoon surface seawater, Korea
C	*Flavobacteriaceae*	*Tenacibaculum*	1.730-1.745	1.66	*T. adriaticum* strain B390	97.2	bryozoan, Adriatic Sea
D	*Psychromonadaceae*	*Psychromonas*	1.715-1.730	0.53	*Ps. japonica* strain JAMM 0394	98.4	marine sediment, Japan
E	*Colwelliaceae*	*Colwellia*	1.730-1.745	2.31	*C. meonggei* strain MA1-3	96.3	sea squirt, South Sea, South Korea
F	*Marinomonadaceae*	*Marinomonas*	1.715-1.730	0.57	*M. profundimaris* strain 25BN12M-4	98.6	deep-sea sediment, Arctic Ocean
G	*Thiotrichaceae*	*Leucothrix*	1.720-1.735	0.75	*L. pacifica* strain XH122	97.7	surface seawater, South Pacific Gyre

^a^buoyant density windows used for differential analysis.

^b^log_2_ fold-change of relative OTU abundance in “heavy” gradient fractions of ^13^C-enriched samples compared to corresponding fractions of ^12^C-natural samples

### Screening and analysis of metagenomic libraries

**Fig. 6:**
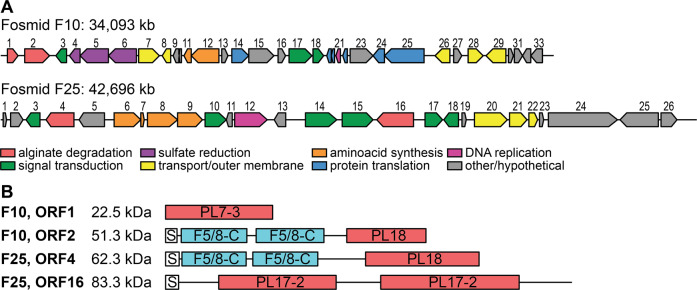
Sequence analysis of fosmid inserts. **A** Genomic map of the DNA insert of the two fosmids F10 and F25 for which positive alginolytic activity was detected. ORFs are numbered sequentially and colored according to predicted function. **B**. Molecular weight and modular analysis of the four predicted alginate lyases in fosmid inserts. S Sec signal peptide type 1, F5/8-C F5/8 type C domain, PL polysaccharide lyase family (CAZy classification) with subfamily when available.

A metagenomic library consisting of 5000 fosmid clones with ~40 kb insert was prepared using DNA obtained from alginate-amended microcosms, representing ~200 Mb of total screened DNA. Using Chao1 index as a proxy for the number of species (average value 123 in alginate-amended microcosms, see Figure S1) and an average genome size of 4 Mb, we can roughly estimate that our screening effort represents 40% of the total community. Two clones showed an alginolytic activity, corresponding to fosmids F10 and F25. Analysis of the fosmid sequences revealed that F10 and F25 contained metagenomic inserts of ~34.1 and 42.7 kb, respectively, encoding 33 and 26 open reading frames (ORFs) in both orientations (Fig. 6A). Both inserts had a homogeneous GC content of 40%. All but one ORF in the F10 insert showed high sequence similarity with proteins from *Alteromonadales* isolates (i.e. *Gammaproteobacteria*, Table S3). In particular, 31/33 ORFs in F10 had their best blastp hits with *Psychromonas* isolates. Similarly, 21/26 ORFs of the F25 metagenomic insert were related to *Alteromonadales*, in particular to *Colwellia* isolates. Both inserts contained two predicted alginate lyase ORFs (ORFs 1 and 2 in F10, ORFs 4 and 16 in F25) together with ORFs related to signal transduction, transport or outer membrane proteins, amino acid synthesis, and DNA replication (Fig. 6B). In addition, F10 insert encoded ORFs related to assimilatory sulfate reduction and protein translation. Modular analysis showed that F10 ORF1 encoded a predicted 22.5 kDa cytoplasmic alginate lyase of the polysaccharide lyase family PL7, subfamily 3. ORF 2 from F10 and ORF 4 from F25 both encoded secreted multimodular alginate lyases consisting of two F5/8 type C domains and a C-terminal PL18 catalytic domain. Despite this similar architecture, ORF 2 and 4 only shared 32% protein sequence identity. Finally, ORF16 from F25 encoded a predicted 83.3 kDa secreted alginate lyase comprising two PL17 domains of subfamily 2.

## Discussion

Estimating the quantitative role of specific heterotrophic bacteria for alginate degradation is essential to constrain carbon budgets in macroalgae-dominated coastal habitats. Here, alginate amendments to the DOC pool of coastal seawater decreased the bacterial diversity in a few hours and favored the growth of a limited number of *Flavobacteriaceae* and *Gammaproteobacteria* OTUs. Combined HISH-SIMS and DNA-SIP analyses provided evidence of alginate assimilation at the single-cell level, directly linking bacterial metabolic functioning to taxonomic identity. The average incorporation rate (~5.89 fg C µm^−3^ h^−1^) was 10-fold higher than that recently measured for phytoplankton-derived DOC uptake by *Flavobacteriales* and *Rhodobacteraceae* cells (~0.42 fg C µm^−3^ h^−1^) [39]. These higher rates might partly be due to the increased alginate availability in microcosms compared to natural environments. They might also highlight the adaptation of the detected ^13^C incorporators to efficient utilization of alginate pulses in coastal seawater. In addition, it underlines the quantitative importance of alginate utilizers for carbon cycling in coastal regions, both for bacterial secondary production and remineralization. Assuming a complete utilization of alginate during the 2-day experiment (Fig. 1), we can extrapolate that maximum 12% of the alginate carbon was assimilated into bacterial biomass, the remaining 88% being respired to CO_2_. This fits previous estimates of carbon flow for kelp mucilage and debris [75, 76], showing bacterial conversion efficiencies of 11–27%. Yet, the large dispersion observed for alginate incorporation rates by *Flavobacteriales* cells (Fig. 4) suggests that they adopt variable nutrient acquisition strategies, while it seems more homogeneous for gammaproteobacterial alginate consumers. This confirms the high degree of niche specialization within closely related clades of marine *Bacteroidetes* [77, 78].

DNA-SIP revealed that only a few initially rare taxa incorporated ^13^C from alginate into their biomass. The small number of detected alginate incorporators might partially reflect (i) the relatively low isotopic ratio of the alginate (3.6 ^13^C At%), compared to traditional SIP experiments with highly labeled substrates and (ii) the high specificity of MW-HR-SIP analysis compared to other DNA-SIP methods [65]. We cannot exclude the possibility that other OTUs enriched in alginate-amended microcosms utilized the substrate, though not enough to be detected using DNA-SIP. However, the proportion of detected alginate incorporators (7/1074 OTUs, i.e., 0.65%) matches other MW-HR-SIP studies on the assimilation of cellulose (63/5940 OTUs, 1.06%) or xylose (49/5940 OTUs, i.e., 0.82%) by soil bacteria [54]. Furthermore, estimation of total carbon assimilation by the seven incorporating OTUs matches the upper limit of bulk measurements (~445 µg C L^−1^ d^−1^), indicating DNA-SIP likely accounted for most quantitatively relevant alginate-assimilating bacteria. Therefore, the small number of detected alginate incorporators in coastal seawater rather indicates that alginolytic systems are not phylogenetically widespread and that polysaccharide availability only selects specialized taxa with distinct ecological niches, as suggested previously [29, 32, 79]. Alginate-assimilating bacteria might be more abundant on algal surfaces, where substrates are constantly present at higher concentration [16]. The alphaproteobacterial SAR11 and flavobacterial NS5 clades dominated the initial bacterial community. Both clades are ubiquitous in open ocean and coastal waters worldwide [78, 80, 81]. They generally feature small genomes of ~2 Mb, which might minimize their metabolic requirements and allow their growth in nutrient-depleted environments [82–84]. The NS5 marine group responds positively to phytoplankton blooms [85] and some NS5 metagenome-assembled genomes feature alginate PULs [86]. Yet, our data suggest they were outcompeted by initially rare copiotrophic taxa that grew rapidly by exploiting the transient increase in alginate. These efficient and fast-growing alginate incorporators notably belonged to *Wenyingzhuangia*, *Polaribacter* and *Tenacibaculum* (*Flavobacteriaceae*), and *Psychromonas* and *Colwellia* (*Alteromonadales*). This corroborates previous findings on cultivated strains that showed alginate-degrading activities in these genera [17, 87–89]. Recent studies also identified alginate-degrading enzymes and PULs in metagenome-assembled genomes related to *Polaribacter*, *Colwellia*, *Psychromonas,* and *Tenacibaculum* in seawater above macroalgal forests [90] or during spring phytoplankton bloom [86]. Here, functional screening of metagenomic libraries confirmed *Alteromonadales* representatives were among the major alginate degraders and featured complementary alginate lyases from different CAZy families. PL7-3 and PL17-2 families are widespread in alginolytic bacteria and comprise endoguluronate lyases [66] and exolytic oligoalginate lyases [91], respectively. By contrast, PL18 alginate lyases seem specific to *Proteobacteria*, as suggested by the lack of reported family members in any *Bacteroidetes* on the CAZy database (http://www.cazy.org/PL18_bacteria.html). The N-terminal pairs of F5/8 type C domain, also known as carbohydrate-binding module of family 32, likely influence the catalytic activity, enzyme stability, or substrate binding as shown for other alginate lyases [92–94]. In both fosmid inserts, alginate lyase genes were close to *ilv* genes involved in branched amino acid biosynthesis (valine, leucine, isoleucine). Since these pathways incorporate pyruvate, an end-product of alginate degradation [95], the colocalization of alginate lyase and *ilv* genes might indicate an important metabolic route for carbon assimilation from alginate.

Directly linking alginate assimilation to a few *Flavobacteriaceae* and *Alteromonadales* strengthens previous studies on the effect of alginate amendments on community composition. Wietz et al. [32] showed that the addition of 0.001% soluble alginate to seawater from the Patagonian continental shelf induced a strong increase in *Alteromonadaceae* that could reach 80% final relative abundance. Similar alginate amendment to Arctic seawater favored a few *Bacteroidia* and *Gammaproteobacteria*, including *Polaribacter* and *Colwellia* [30]. Alginate particles added to coastal surface seawater from California [31] or Massachusetts [29] also induced the growth of *Bacteroidetes* and *Alteromonadales*, including *Psychromonas*. Therefore, it appears that closely related taxa respond to alginate in distant, contrasting environments. Their success for polysaccharide degradation relies on numerous PULs targeting diverse algal compounds, including alginate. The tight control of flavobacterial PUL expression that allows overexpression of alginolytic genes within minutes after alginate becomes available [24] might explain the rapid substrate exploitation in microcosms. Recently, a tripartite conceptual model of the different bacterial strategies at play during marine polysaccharide degradation was proposed [96]. *Selfish bacteria* break up polysaccharides and internalize oligomers with virtually no loss of low-molecular-weight products to the environment [34], contrasting with *external hydrolyzers* that use extracellular enzymes and release degradation products to the milieu. The liberated products are subsequently used by *scavenging bacteria*, which cannot or do not produce extracellular enzymes. In this model, both selfish and external hydrolyzers could compete for highly complex polysaccharides present in high abundance and often forming gels [96]. Here, the major flavobacterial alginate incorporators could represent selfish bacteria, owing to their concerted substrate degradation and uptake, archetypal to PULs involving SusC/D-like membrane proteins in *Bacteroidetes* but not in *Gammaproteobacteria* [97]. They may have varying efficiency in assimilating degradation products, as suggested by their large range of incorporation rates. Conversely, the *Alteromonadales* incorporators likely represent external hydrolyzers, as recently proposed in a study of substrate utilization in the Atlantic Ocean where “sharing” organisms belonged mostly to *Alteromonadaceae* [98]. The fact that three of the four predicted alginate lyases encoded in the fosmid inserts feature signal peptides supports this hypothesis. The other detected alginate incorporators (i.e. *Marinomonas* and *Leucothrix*) might be scavengers, relying on previous degradation by other microorganisms and therefore not showing the same rapid increase in relative abundance. Indeed, *Marinomonas* isolates generally do not degrade polymeric alginate [99] and metatranscriptomics of coastal seawater suggested *Oceanospirillales* are efficient scavengers of monosaccharides from DOM [100]. Investigation of isotope incorporation at successive time points would help disentangle the interactions between alginate consumers with different strategies, and decipher the dynamics of carbon transfer via the microbial loop. Furthermore, our work paves the way for isotopic tracing studies using purified macroalgal compounds, exudates, or intact tissues to identify (1) the substrate niche(s) of marine bacteria and (2) trophic chains based on utilization of specific substrates *via* diverse detrital pathways, leading thus to better characterization and quantification of their contributions to the coastal carbon cycle.

## Supplementary information

Supplementary Information

Table S1

Table S2

Table S3

Figure S1

Figure S2

Figure S3

Figure S4
