# Supplementary material for: Isotopic tracing reveals single-cell assimilation of a macroalgal polysaccharide by a few marine Flavobacteria and Gammaproteobacteria
Source: ISME J. 2021 May 5;15(10):3062–75. doi: 10.1038/s41396-021-00987-x (PMC8443679; doi:10.1038/s41396-021-00987-x)
Supplement: Supplementary file 1 — Supplementary Information [file 41396_2021_987_MOESM1_ESM.docx]

**Isotopic tracing reveals single-cell assimilation of a macroalgal polysaccharide by a few marine Flavobacteria and Gammaproteobacteria**

François Thomas*^1^, Nolwen Le Duff^1^, Ting-Di Wu^2,3^, Aurélie Cébron^4^, Stéphane Uroz^5^, Pascal Riera^6^, Cédric Leroux^7^, Gwenn Tanguy^8^, Erwan Legeay^8^, Jean-Luc Guerquin-Kern^2,3^

**^1^** Sorbonne Université, CNRS, Integrative Biology of Marine Models (LBI2M), Station Biologique de Roscoff (SBR), 29680 Roscoff, France

**^2^** Institut Curie, Université Paris-Saclay, Paris, France

**^3^** Université Paris-Saclay, INSERM US43, CNRS UMS2016, Multimodal Imaging Center, Orsay, France

**^4^** Université de Lorraine, CNRS, LIEC, F-54500 Nancy, France

**^5^** Université de Lorraine, INRAE, UMR1136 « Interactions Arbres-Microorganismes », F-54280 Champenoux, France

**^6^** Sorbonne Université, CNRS, UMR7144, Station Biologique de Roscoff (SBR), 29680 Roscoff, France

^7^ CNRS, Sorbonne Université, FR2424, Metabomer, Station Biologique de Roscoff, 29680 Roscoff, France

**^8^** CNRS, Sorbonne Université, FR2424, Genomer, Station Biologique de Roscoff, 29680 Roscoff, France

* Correspondence to francois.thomas@sb-roscoff.fr

**SUPPLEMENTARY INFORMATION**

**Supplementary methods**

*Production of ^13^C-enriched alginate*

The production of ^12^C-natural and ^13^C-enriched alginate was based on a recently developed protocol [1]. Two batches of sporophytes of the brown macroalga *Laminaria digitata* were cultivated in controlled conditions with regular amendments of either NaHCO_3_ with natural isotopic composition (98.9 ^12^C%) or labeled NaHCO_3_ (99 ^13^C%), respectively. Algae were retrieved after three months, dried at 40°C for 2 days and pulverized in a MM200 mixer mill (Retsch) for 90 s at 30 Hz. Alginate was extracted from ca. 10 g of algal powder from each batch separately, using a chemical extraction. The powder was resuspended in sequential ethanol baths with increasing concentration (70%, 80%, 96%), followed by successive rinses in methanol/chloroform 50:50 (v/v) and acetone. The obtained alcohol-insoluble residues were mixed in 2% (w/v) CaCl_2_ for 4 h at 80°C. The calcium-insoluble pellet containing alginate was retrieved by centrifugation (15 min, 10,000 rpm) and resuspended in 4% Na_2_CO_3_ for 2 h at 80°C, followed by centrifugation (15 min, 10,000 rpm). The supernatant was adjusted to pH 2 with sulfuric acid to eliminate carbonate and precipitate alginic acid, which was further resuspended in distilled water and neutralized with NaOH. Finally, sodium alginate was retrieved by ethanol precipitation, lyophilized and ground for long-term storage.

*HISH-SIMS imaging of single cells*

High spatial resolution SIMS imaging was performed using a NanoSIMS-50 Ion microprobe (CAMECA, Gennevilliers, France) operating in scanning mode [2, 3]. Prior to being loaded into the NanoSIMS-50, a 4-mm diameter section was carefully extracted from each filter with a punch. The sections were then inserted between a mask with 4 holes of 3-mm each (4-hole thin plate, part #45620694, CAMECA) initially designed for receiving standard 3-mm TEM grids, and a large flat stainless steel cylinder of 10-mm in diameter underneath. Analyses were performed using a tightly focused Cs^+^ primary ion beam at an impact energy of 16 keV. First, Cs^+^-ion pre-implantation was carried out to partially sputter away the agarose layer embedding the cells. At the same time, this helps to achieve efficient ion emission yield for the negative secondary ions [2]. Then, five ion species, ^12^C^-^, ^19^F^-^, ^12^C^14^N^-^, ^13^C^14^N^-^ and ^32^S^-^, were monitored in parallel from the same sputtered volume, and the images for these selected ion species were generated by stepping the primary beam over the surface of the sample. Prior to high spatial resolution acquisition, large areas were surveyed to detect regions of interest. For high-resolution imaging, the primary beam intensity was 1.5 pA with a typical probe size of ≈ 150 nm. The raster size was either 12 or 24 μm with an image definition of 256x256 pixels and 340×340 pixels, respectively. The acquisition was carried out in multi-frame mode with a dwell time of 0.5 ms per pixel and 30 to 180 frames were recorded for each image field. Images were processed using ImageJ [4]. Successive image frames were properly aligned using TomoJ [5] with ^12^C^14^N^-^ images as reference to correct the slight image shift during time of signal accumulation. As an example, for the analysis of Fig 3 G-H-I, the field of view was 24 µm and the image definition was 340 x 340 pixels. With a dwell time of 0.5 ms per pixel and an accumulation of 180 frames, the duration of acquisition was 2h53min (340x340 x0.5 10^-3^ x180 = 10404 sec). A summed image was obtained for each ion species. The image of ^32^S^-^ shows the biomass of the investigated area. In addition, as the ^32^S^-^ emission is quite homogeneous for different cells, it was further employed to generate ROIs (Region of Interest) for individual cells. In the raw image of ^19^F^-^, targeted cells exhibited much higher signal although a variable background level remained detectable. To compare ^19^F^-^ ion intensity from one acquisition to another, we normalized the signal with respect to ^32^S^-^ as [^19^F^-^]/[^32^S^-^]. We would like to emphasize that a background signal was generally observed when detecting ^19^F^-^ species (see for example **Figure S3**). Thus, throughout the imaging process, care was taken to keep such background level as low as possible. The Cs^+^-implantation results in high Cs concentration on sample surface that becomes very reactive and acquires a high affinity to halogen compounds, such as fluorine-containing molecules in the vacuum of the instrument (either from cleaning agents for metallic or ceramic parts, or from various insulators, as Teflon tubes). Therefore, ultra-high vacuum environment at a pressure of 6 10^-11^ mbar (i.e. 6 10^-9^ Pa) was maintained in the analytical chamber during NanoSIMS imaging. In addition, continuous bombardment of the analyzed area by rapid scanning of the primary ion beam is mandatory in order to avoid accumulation of residual gas. Consequently, the image acquisition was carried out in multi-frame mode immediately after the pre-implantation.

The ^13^C atom fraction map was established from ^12^C^14^N^-^ and ^13^C^14^N^-^ images based on pixel-by-pixel calculation as follows:

^13^C At% = [^13^C^14^N^-^]/([^12^C^14^N^-^]+[^13^C^14^N^-^]) x 100%

For a selected cell, the average ^13^C at% can be computed by taking the accumulated counts inside the whole ROI for higher precision, in contrast with individual pixel-to-pixel value displaying much larger statistical fluctuation. The final ^13^C at% map was displayed using Hue-Saturation-Intensity (HSI) transformation. These HSI color images were generated using OpenMIMS, an ImageJ plugin developed by Claude Lechene’s laboratory [6]. The hue corresponds to the at% value, and the intensity at a given hue is an index of the statistical reliability.

Cell-free filter areas were used as an internal check of the unlabeled ^13^C composition of the sample. From 13 acquisitions spread over several months, the measured mean [^13^C^14^N^-^]/([^12^C^14^N^-^] ratio value was 0.01136 +/-0.00014 (n = 24).

Single-cell carbon assimilation rates were inferred from NanoSIMS data, following calculations developed in Stryhanyuk et al., 2018 [7].

The measured isotope ratios R' were corrected for C dilution due to the CARD-FISH treatment [8, 9] as follows:

$$R= \frac{R^{'}+ K_{i}\times\left( R^{'}- D_{ch} \times\left( R^{'}+1 \right) \right)}{1- K_{i} \times\left( R^{'}- D_{ch} \times\left( R^{'}+1 \right) \right)}$$

where *R'* is the observed isotope ratio, *K_i_* is the fraction of introduced carbon due to chemical treatments and *D_ch_* is the fraction of ^13^C in chemicals used during the procedure. We assumed *D_ch_* = 0.011, corresponding to the natural ^13^C abundance. We used a *K_i_* value of 29.38%, previously measured for *E. coli* cells grown with 6% ^13^C labeled D-glucose and processed with a similar procedure as the one we used [10]. The fraction of carbon assimilated by a cell during growth with ^13^C-enriched alginate was calculated as follows:

$$K_{A}= \frac{R_{f}- R_{i}}{R_{i}+1} \times\frac{R_{gs}+1}{R_{gs}- R_{f}}$$

where *K_A_* is the fraction of assimilated carbon, *R_f_* is the isotope ratio after incubation, *R_i_* is the initial cellular isotope ratio before incubation (assumed to be natural ^13^C abundance) and *R_gs_* is the isotope ratio of growth substrate during incubation (here *R_gs_* = 0.03764, corresponding to ^13^C-enriched alginate at 3.6279 At%^13^C). The length and width of ROI-confined fragments of cells were expressed as follows:

$$L= \sqrt{\frac{S_{p}}{LWR-1+ \frac{\pi}{4}}} \times LWR \times\frac{FoV}{Rst}$$

$$W= \sqrt{\frac{S_{p}}{LWR-1+ \frac{\pi}{4}}} \times\frac{FoV}{Rst}$$

where *L* and *W* are the length and width in metric scale, respectively, *Sp* is the ROI area in pixels, *LWR* is the ROI length-to-width ratio, *FoV* is the length of rectangular raster in µm and *Rst* is the raster size in pixels. Cell biovolume *V* (in µm^3^) was calculated as follows:

$$V= \frac{1}{2}\pi\times W^{2} \times\left( \frac{1}{3}W+ \frac{1}{2}\left( L-W \right) \right)$$

Final volume-specific carbon assimilation rates were calculated as follows:

$$F_{V}= \frac{\rho_{V} \times K_{A}}{t}$$

where *F_V_* is the assimilation rate per unit of cell volume (in fg µm^-3^ h^-1^), *ρ_V_* is the partial density of carbon in cells (in fg µm^-3^) and *t* is the incubation time (in hours). We used a partial density of carbon in bacterial cells of 111.9 ± 45.5 fg µm^-3^ [11].

*16S rRNA gene metabarcoding analysis*

16S rRNA gene metabarcoding library preparation and sequencing was performed as described previously [12]. A 464-bp fragment of 16S rRNA genes was amplified from non-fractionated total DNA samples [T0 (n = 3), ^12^C-natural alginate (n = 3), ^13^C-enriched alginate (n = 3) and unamended controls (n = 3)] and gradient fractions (n = 14 fractions per gradient) using primers S-D-Bact-0341-b-S-17 (5'-CCTACGGGNGGCWGCAG-3') and S-D-Bact-0785-a-A-21 (5'-GACTACHVGGGTATCTAATCC-3') [13], in 12.5 µl reactions containing 0.5 ng of DNA, 500 nM of each primer and 1X Master Mix Q5 (New England Biolabs). PCR was performed by heating reactions for 3 min at 95°C, followed by 25 cycles of 30 s at 95°C, 30 s at 55°C, 30 s at 72°C, and a final elongation step of 5 min at 72°C. Amplicons were purified using AMPure XP beads (Beckman-Coulter, Brea, USA), attached to dual indices and Illumina adapters using the Nextera XT Index kit, analyzed on a Labchip GXT (Perkin Elmer, USA) and pooled at equimolar concentrations. The library was sequenced using a MiSeq Reagent kit v3 (2 x 300 cycles, Illumina) at the Genomer Platform (Station Biologique de Roscoff, France). MiSeq sequencing yielded a total of 7,250,023 paired-end sequences of 300 bp length from 96 samples. Reads were truncated at 265 bp to remove low-quality ends. Reads R1 and R2 were trimmed at 17 bp and 21 bp, respectively, to remove primer sequences. Paired-end reads were merged, denoised and checked for chimeras using default parameters of DADA2 [14] implemented in QIIME 2 v2018.8 [15]. Denoised sequences were clustered *de novo* in OTUs at 97% using vsearch [16]. Taxonomy was assigned to a representative sequence for each OTU using the sklearn classifier trained on the SILVA ssu132 Ref NR99 database trimmed to the amplified region. A phylogenetic tree was created with aligned representative sequences using FastTree in QIIME 2. OTUs affiliated to chloroplasts, mitochondria or archaea and OTUs representing less than 0.001% of total sequences were discarded (representing 2.6% of input sequences). The final dataset comprised 1,074 OTUs for a total of 2,909,992 sequences, ranging from 12,948 to 174,305 sequences per sample. Data were further analyzed in phyloseq R package [17]. Alpha-diversity indices for non-fractionated samples were calculated on the non-transformed dataset. Beta-diversity analyses were performed on Hellinger-transformed datasets using PCoA or NMDS on a weighted Unifrac distance matrix. The effect of alginate amendments on total community structure was tested using PERMANOVA with 999 permutations. Differential abundance analysis between unamended controls (n = 3) and alginate-amended microcosms (n = 6) was performed using DESeq2 [18] based on the negative binomial distribution with Wald test for significance and parametric fitting. Differences were considered significant when Benjamini-Hochberg corrected p-values were lower than 5% and log2 fold-change > 1.

**Supplementary table list**

**Table S1**: Final proportions (T = 47 h) of *Flavobacteriales* and *Gammaproteobacteria* in total communities of triplicate incubations with ^13^C-enriched alginate, obtained by CARD-FISH and metabarcoding of the 16S rRNA gene (MetaB).

**Table S2**: Summary of NanoSIMS data analysis.

**Table S3:** Functional gene annotation of fosmid inserts.

**Supplementary figure legends**

**Figure S1:** Alpha- and beta-diversity analysis for initial bacterial communities (T0) and after 47 h in unamended microcosms (T47-CTRL) or microcosms amended with either ^12^C-natural (T47-ALG12) or ^13^C-enriched alginate (T47-ALG13). Results were obtained from non-fractionated DNA samples. **A.** Richness and diversity indices. **B.** PCoA plot based on a weighted Unifrac distance matrix for Hellinger-transformed OTU-level datasets. The first two principal coordinates axes and percentage of variation explained are shown.

**Figure S2:** Mean log_2_ fold-change of OTUs showing a significant change in sequence relative abundance between alginate-amended microcosms (n = 6) and unamended microcosms (n = 3) after 47 h. Each dot represents a single OTU, classified at the genus level and colored according to its taxonomic class. A positive log_2_ fold-change denotes a higher relative abundance in alginate-amended microcosms compared to unamended control microcosms. unc.:unclassified.

**Figure S3**: NanoSIMS analysis of cells hybridized with the non-sense probe. **A.** Secondary ion image of ^32^S (a.u: arbitrary intensity unit). **B.** Ratio ^19^F/^32^S. Scale bar: 2 µm.

**Figure S4:** Detection of ^13^C-incorporating OTUs using MW-HRSIP. **A.** Relative sequence abundance of OTUs as a function of the fraction buoyant density for ^12^C-natural (black) or ^13^C-enriched (red) alginate amended microcosms. Values are mean ± s.e.m. (n = 3, except replicate T47-ALG13-1 was omitted for OTU A in ^13^C-enriched condition to avoid over-representation of *Wenyingzhuangia*). The heavy window used for MW-HRSIP approach is depicted in grey for each OTU. **B.** Relative sequence abundance in the heavy window for ^13^C-incorporating OTUs. Values are mean ± s.e.m. (n = 3), depicted on a log scale.

**References for Supplementary Methods**

1. Thomas F, Le Duff N, Leroux C, Dartevelle L, Riera P. Isotopic labeling of cultured macroalgae and isolation of ^13^C-labeled cell wall polysaccharides for trophic investigations. *Adv Bot Res* 2020; **95**: 1–17.

2. Guerquin-Kern JL, Wu T Di, Quintana C, Croisy A. Progress in analytical imaging of the cell by dynamic secondary ion mass spectrometry (SIMS microscopy). *Biochim Biophys Acta - Gen Subj* 2005; **1724**: 228–238.

3. Slodzian G, Daigne B, Girard F, Boust F, Hillion F. Scanning secondary ion analytical microscopy with parallel detection. *Biol Cell* 1992; **74**: 43–50.

4. Schneider CA, Rasband WS, Eliceiri KW. NIH Image to ImageJ: 25 years of Image Analysis. *Nat Methods* 2012; **9**: 671–675.

5. MessaoudiI C, Boudier T, Sorzano COS, Marco S. TomoJ: Tomography software for three-dimensional reconstruction in transmission electron microscopy. *BMC Bioinformatics* 2007; **8**: 1–9.

6. Lechene C, Hillion F, McMahon G, Benson D, Kleinfeld A, Kampf JP, et al. High-resolution quantitative imaging of mammalian and bacterial cells using stable isotope mass spectrometry. *J Biol* 2006; **5**: 20.

7. Stryhanyuk H, Calabrese F, Kümmel S, Musat F, Richnow HH, Musat N. Calculation of single cell assimilation rates from sip-nanosims-derived isotope ratios: A comprehensive approach. *Front Microbiol* 2018; **9**: 1–15.

8. Musat N, Stryhanyuk H, Bombach P, Adrian L, Audinot JN, Richnow HH. The effect of FISH and CARD-FISH on the isotopic composition of ^13^C- and ^15^N-labeled *Pseudomonas putida* cells measured by nanoSIMS. *Syst Appl Microbiol* 2014; **37**: 267–276.

9. Meyer N, Fortney JL, Dekas AE. NanoSIMS sample preparation decreases isotope enrichment: magnitude, variability and implications for single-cell rates of microbial activity. *Environ Microbiol* 2021; **23**: 81-98.

10. Woebken D, Burow LC, Behnam F, Mayali X, Schintlmeister A, Fleming ED, et al. Revisiting N_2_ fixation in Guerrero Negro intertidal microbial mats with a functional single-cell approach. *ISME J* 2015; **9**: 485–496.

11. Arandia-Gorostidi N, Alonso-Sáez L, Stryhanyuk H, Richnow HH, Morán XAG, Musat N. Warming the phycosphere: Differential effect of temperature on the use of diatom-derived carbon by two copiotrophic bacterial taxa. *Environ Microbiol* 2020; **22**: 1381–1396.

12. Thomas F, Dittami SM, Brunet M, Le Duff N, Tanguy G, Leblanc C, et al. Evaluation of a new primer combination to minimize plastid contamination in 16S rDNA metabarcoding analyses of alga‐associated bacterial communities. *Environ Microbiol Rep* 2020; **12**: 30–37.

13. Klindworth A, Pruesse E, Schweer T, Peplies J, Quast C, Horn M, et al. Evaluation of general 16S ribosomal RNA gene PCR primers for classical and next-generation sequencing-based diversity studies. *Nucleic Acids Res* 2013; **41**: 1–11.

14. Callahan BJ, McMurdie PJ, Rosen MJ, Han AW, Johnson AJA, Holmes SP. DADA2: High-resolution sample inference from Illumina amplicon data. *Nat Methods* 2016; **13**: 581–583.

15. Bolyen E, Rideout JR, Dillon MR, Bokulich NA, Abnet CC, Al-Ghalith GA, et al. Reproducible, interactive, scalable and extensible microbiome data science using QIIME 2. *Nat Biotechnol* 2019; **37**: 852–857.

16. Rognes T, Flouri T, Nichols B, Quince C, Mahé F. VSEARCH: A versatile open source tool for metagenomics. *PeerJ* 2016; **4**: e2584.

17. Mcmurdie PJ, Holmes S. phyloseq : An R package for reproducible interactive analysis and graphics of microbiome census data. *PLoS One* 2013; **8**: 1–11.

18. Love MI, Huber W, Anders S. Moderated estimation of fold change and dispersion for RNA-seq data with DESeq2. *Genome Biol* 2014; **15**: 1–21.
