# Supplementary material for: Isotopic tracing reveals single-cell assimilation of a macroalgal polysaccharide by a few marine Flavobacteria and Gammaproteobacteria
Source: ISME J. 2021 May 5;15(10):3062–75. doi: 10.1038/s41396-021-00987-x (PMC8443679; doi:10.1038/s41396-021-00987-x)
Supplement: Supplementary file 2 — Table S1 [file 41396_2021_987_MOESM2_ESM.docx]

**Table S1**: Final proportions (T = 47 h) of *Flavobacteriales* and *Gammaproteobacteria* in total communities of triplicate incubations with ^13^C-enriched alginate, obtained by CARD-FISH and metabarcoding of the 16S rRNA gene (MetaB).

|  | *Flavobacteriales* (CF319a) | |  | *Gammaproteobacteria*  (GAM42a) | |
| --- | --- | --- | --- | --- | --- |
|  | CARD-FISH^a^ | MetaB |  | CARD-FISH | MetaB |
| T47-ALG13-1 | 0.34 | 0.4 |  | 0.27 | 0.47 |
| T47-ALG13-3 | nd | 0.17 |  | 0.42 | 0.66 |
| T47-ALG13-2 | 0.16 | 0.19 |  | 0.66 | 0.62 |
| mean ± sem | 0.25±0.09 | 0.25±0.07 |  | 0.45±0.11 | 0.58±0.06 |
| ^a^ Values for CARD-FISH are proportion of DAPI-stained cells. Twenty-six microscopic fields were analyzed per sample, amounting 358-1842 DAPI-stained cells. ^b^ Values for metabarcoding are relative sequence abundance of 16S rRNA reads. nd: not determined (filter lost) | | | | | |
