# Supplementary material for: Isotopic tracing reveals single-cell assimilation of a macroalgal polysaccharide by a few marine Flavobacteria and Gammaproteobacteria
Source: ISME J. 2021 May 5;15(10):3062–75. doi: 10.1038/s41396-021-00987-x (PMC8443679; doi:10.1038/s41396-021-00987-x)
Supplement: Supplementary file 3 — Table S2 [file 41396_2021_987_MOESM3_ESM.pdf]

Table S2: Summary of HISH-SIMS data analysis.

Acquisition	ROI#	Probe	HISH signal	FoV length of square raster in μm	Rst raster size in pixels	Sp ROI area in pixel^2	LWR length-to-width ratio of ROI	Wp in pixel	Lp in pixel	W width in μm	L length in μm	V cell volume in μm^3	R measured (R')	R corrected (R)	Atom Percent Enrichment	K_A	m mass of carbon in each cell (fg)	u mass of carbon assimilated by each cell (fg)	Fc cell-specific assimilation rate (fg.cell^{-1}.h^{-1})	Fv volume-specific assimilation rate (fg.μm^{-3}h^{-1})
Jun20_18th_#2-1_4pm15	1	CF319a	+	12	256	320	2.92	10.88	31.76	0.51	1.49	0.27	0.0276	0.0325	2.0484	4.2718	30.1205	128.6699	2.7377	10.1706
Jun20_18th_#2-1_4pm15	2	CF319a	+	12	256	311	3.92	9.16	35.91	0.43	1.68	0.22	0.0267	0.0314	1.9453	3.3388	24.9645	83.3519	1.7734	7.9492
Jun20_18th_#2-1_6pm	1	CF319a	+	12	256	620	10.2	7.88	80.37	0.37	3.77	0.39	0.0270	0.0318	1.9814	3.6260	43.6972	158.4461	3.3712	8.6330
Feb20_12th#1-1_10am30	3	CF319a	+	26	300	634	8.62	8.68	74.86	0.88	6.49	3.79	0.0244	0.0284	1.6637	1.9250	423.5475	815.3478	17.3478	4.5832
Jun20_11th_#2-1_11am	1	CF319a	+	12	256	297	3.12	10.11	31.54	0.47	1.48	0.23	0.0247	0.0287	1.6905	2.0187	26.0708	52.6304	1.1198	4.8063
Jun20_11th_#2-1_11am	2	CF319a	+	12	256	90	1.94	7.22	14.01	0.34	0.66	0.05	0.0127	0.0131	0.1963	0.0842	5.4790	0.4613	0.0098	0.2005
Jun20_11th_#2-1_11am	3	CF319a	+	12	256	113	3.73	5.67	21.15	0.27	0.99	0.05	0.0131	0.0137	0.2476	0.1086	5.6034	0.6084	0.0129	0.2585
Jun20_16th_#2-1_8pm	1	CF319a	+	24	320	301	1.42	15.80	22.44	1.48	1.68	2.05	0.0292	0.0347	2.2510	8.1282	229.3628	1864.3070	39.6661	19.3520
Jun20_16th_#2-1_8pm	2	CF319a	+	24	320	331	2.9	11.10	32.20	1.04	2.41	1.76	0.0276	0.0325	2.0470	4.2569	196.8737	838.0685	17.8312	10.1350
Jun20_16th_#2-1_8pm	3	CF319a	+	24	320	238	3.65	8.32	30.38	0.78	2.28	0.97	0.0202	0.0228	1.1333	0.8127	108.0125	87.7784	1.8676	1.9348
Jun20_16th_#2-1_8pm	4	CF319a	+	24	320	147	1.11	12.81	14.22	1.20	1.07	0.76	0.0114	0.0114	0.0318	0.0128	84.4924	1.0779	0.0229	0.0304
Jun20_18th_#2-1_8pm30	1	CF319a	+	12	256	382	4.08	9.94	40.56	0.47	1.90	0.30	0.0281	0.0332	2.1123	5.0831	33.3190	169.3639	3.6035	12.1021
Jun20_18th_#2-1_10pm30	1	CF319a	+	12	256	137	7.63	4.30	32.80	0.20	1.54	0.05	0.0244	0.0284	1.6636	1.9247	5.2450	10.0952	0.2148	4.5825
Jun20_18th_#2-1_10pm30	2	CF319a	+	12	256	98	3.79	5.24	19.84	0.25	0.93	0.04	0.0247	0.0287	1.6938	2.0306	4.4901	9.1177	0.1940	4.8346
Jun20_18th_#2-1_10pm30	3	CF319a	+	12	256	98	2.38	6.73	16.01	0.32	0.75	0.05	0.0249	0.0290	1.7193	2.1263	5.6406	11.9935	0.2552	5.0624
Jun20_18th_#2-1_10pm30	4	CF319a	+	12	256	62	2.58	5.12	13.21	0.24	0.62	0.02	0.0237	0.0275	1.5750	1.6527	2.7291	4.5104	0.0960	3.9349
Jun20_18th_#2-1_10pm30	5	CF319a	+	12	256	204	2.05	10.54	21.61	0.49	1.01	0.16	0.0244	0.0284	1.6621	1.9198	18.2087	34.9579	0.7438	4.5709
Jun20_18th_#2-1_10pm30	6	CF319a	+	12	256	103	2.33	6.98	16.26	0.33	0.76	0.05	0.0139	0.0147	0.3491	0.1602	6.1407	0.9840	0.0209	0.3815
Jun20_18th_#2-1_10pm30	7	CF319a	+	12	256	50	1.48	6.29	9.30	0.29	0.44	0.02	0.0222	0.0255	1.3884	1.2184	2.5781	3.1410	0.0668	2.9007
Jun20_12th_#2-1_3pm	1	CF319a	+	12	256	183	1.72	11.03	18.96	0.52	0.89	0.15	0.0250	0.0292	1.7383	2.2014	16.8235	37.0350	0.7880	5.2412
Jun20_12th_#2-1_3pm	2	CF319a	+	12	256	152	2.28	8.58	19.56	0.40	0.92	0.10	0.0262	0.0307	1.8815	2.9110	11.1249	32.3849	0.6890	6.9307
Jun20_12th_#2-1_3pm	3	CF319a	+	12	256	148	1.94	9.26	17.97	0.43	0.84	0.10	0.0262	0.0307	1.8793	2.8976	11.5539	33.4781	0.7123	6.8987
Jun20_12th_#2-1_3pm	4	CF319a	-	12	256	96	1.77	7.86	13.91	0.37	0.65	0.06	0.0215	0.0246	1.3012	1.0607	6.3059	6.6888	0.1423	2.5255
Jun20_12th_#2-1_3pm	5	CF319a	+	12	256	86	2.13	6.70	14.27	0.31	0.67	0.04	0.0122	0.0125	0.1381	0.0578	4.8929	0.2828	0.0060	0.1376
Jun20_12th_#2-1_3pm	7	CF319a	+	12	256	67	4.4	4.00	17.60	0.19	0.83	0.02	0.0213	0.0243	1.2710	1.0112	2.3577	2.3841	0.0507	2.4075
Jun20_15th_#2-1_7pm15	1	CF319a	+	24	320	143	4.08	6.08	24.82	0.57	1.86	0.43	0.0211	0.0241	1.2560	0.9876	47.7543	47.1604	1.0034	2.3512
Jun20_15th_#2-1_7pm15	2	CF319a	-	24	320	150	2.64	7.86	20.76	0.74	1.56	0.56	0.0290	0.0343	2.2190	7.1842	62.6456	450.0568	9.5757	17.1044
Jun20_15th_#2-1_7pm15	3	CF319a	-	24	320	181	3.38	7.56	25.56	0.71	1.92	0.66	0.0290	0.0344	2.2215	7.2515	74.2300	538.2774	11.4527	17.2647
Jun20_15th_#2-1_7pm15	4	CF319a	-	24	320	117	2.13	7.82	16.65	0.73	1.25	0.42	0.0275	0.0324	2.0367	4.1462	47.3866	196.4733	4.1803	9.8714
Jun20_15th_#2-1_7pm15	5	CF319a	-	24	320	128	2.91	6.89	20.05	0.65	1.50	0.42	0.0286	0.0339	2.1798	6.2628	47.2699	296.0432	6.2988	14.9109
Jun20_15th_#2-1_7pm15	6	CF319a	-	24	320	114	1.93	8.15	15.73	0.76	1.18	0.42	0.0270	0.0317	1.9738	3.5623	47.4972	169.1984	3.6000	8.4813
Jun20_15th_#2-1_7pm15	7	CF319a	-	24	320	133	1.54	10.02	15.43	0.94	1.16	0.58	0.0285	0.0337	2.1583	5.8392	65.4170	381.9808	8.1273	13.9022
Jun20_15th_#2-1_7pm15	8	CF319a	-	24	320	112	3.94	5.48	21.60	0.51	1.62	0.30	0.0268	0.0315	1.9577	3.4332	33.6469	115.5163	2.4578	8.1739
Jun20_15th_#2-1_7pm15	9	CF319a	-	24	320	144	3.44	6.68	22.99	0.63	1.72	0.47	0.0231	0.0267	1.5034	1.4675	52.2491	76.6729	1.6313	3.4938
Mar20_11th_#3-2_6pm	1	GAM42a	-	24	341	423	1.94	15.66	30.38	1.47	2.14	2.79	0.0279	0.0330	2.0912	4.7884	312.1940	1494.9029	31.8064	11.4004
Mar20_11th_#3-2_6pm	2	GAM42a	-	24	341	158	2.56	8.21	21.01	0.77	1.48	0.57	0.0269	0.0316	1.9633	3.4769	63.6054	221.1517	4.7054	8.2781
Mar20_11th_#3-2_6pm	3	GAM42a	-	24	341	74	1.32	8.18	10.80	0.77	0.76	0.23	0.0243	0.0282	1.6413	1.8512	26.0848	48.2885	1.0274	4.4075
Mar20_11th_#3-2_6pm	4	GAM42a	+	24	341	436	3.27	11.95	39.06	1.12	2.75	2.34	0.0261	0.0305	1.8627	2.8003	261.8894	733.3703	15.6036	6.6671
Mar20_11th_#3-2_6pm	5	GAM42a	-	24	341	251	3.01	9.48	28.52	0.89	2.01	1.06	0.0267	0.0314	1.9423	3.3171	118.6914	393.7066	8.3767	7.8974
Mar20_11th_#3-2_6pm	9	GAM42a	+	24	341	361	3.44	10.58	36.39	0.99	2.56	1.72	0.0271	0.0318	1.9866	3.6698	192.8615	707.7562	15.0586	8.7372
Mar20_11th_#3-2_6pm	10	GAM42a	-	24	341	213	2.85	8.99	25.62	0.84	1.80	0.85	0.0268	0.0315	1.9582	3.4373	95.0415	326.6869	6.9508	8.1837
Mar20_11th_#3-2_6pm	11	GAM42a	+	24	341	236	5.63	6.60	37.17	0.62	2.62	0.72	0.0237	0.0274	1.5671	1.6310	81.1098	132.2877	2.8146	3.8831
Mar20_11th_#3-2_6pm	12	GAM42a	-	24	341	319	2.33	12.28	28.61	1.15	2.01	1.70	0.0270	0.0318	1.9834	3.6429	189.8701	691.6751	14.7165	8.6732
Mar20_11th_#3-2_6pm	13	GAM42a	+	24	341	430	2.62	13.37	35.03	1.25	2.47	2.53	0.0265	0.0311	1.9144	3.1204	282.7473	882.2970	18.7723	7.4293
Mar20_11th_#3-2_6pm	14	GAM42a	+	24	341	277	2.2	11.81	25.99	1.11	1.83	1.41	0.0256	0.0299	1.8074	2.5084	157.3220	394.6223	8.3962	5.9721
Mar20_11th_#3-2_6pm	15	GAM42a	+	24	341	246	3.57	8.56	30.57	0.80	2.15	0.95	0.0267	0.0314	1.9450	3.3364	106.6826	355.9374	7.5731	7.9435
Mar20_11th_#3-2_6pm	16	GAM42a	-	24	341	343	3.18	10.75	34.20	1.01	2.41	1.65	0.0246	0.0286	1.6820	1.9883	185.0336	367.8932	7.8275	4.7337
Mar20_12th_#3-2_4pm35	1	GAM42a	+	12	256	342	2.08	13.54	28.16	0.63	1.32	0.35	0.0240	0.0279	1.6148	1.7686	39.2496	69.4164	1.4769	4.2107
Mar20_12th_#3-2_4pm35	2	GAM42a	+	12	256	389	1.73	16.02	27.72	0.75	1.30	0.46	0.0253	0.0295	1.7694	2.3327	51.9961	121.2890	2.5806	5.5537
Mar20_11th#3-2_5pm15	1	GAM42a	+	12	256	164	2.36	8.74	20.63	0.41	0.97	0.11	0.0257	0.0301	1.8196	2.5688	12.2611	31.4959	0.6701	6.1158
Mar20_11th#3-2_5pm15	2	GAM42a	+	12	256	274	3.52	9.10	32.05	0.43	1.50	0.19	0.0266	0.0312	1.9232	3.1807	21.7706	69.2468	1.4733	7.5729
Mar20_11th#3-2_5pm15	3	GAM42a	-	12	256	218	4.09	7.50	30.68	0.35	1.44	0.13	0.0263	0.0308	1.8907	2.9672	14.3469	42.5706	0.9058	7.0646
Mar20_11th#3-2_5pm15	4	GAM42a	-	12	256	84	4.35	4.51	19.61	0.21	0.92	0.03	0.0215	0.0246	1.2985	1.0563	3.3285	3.5158	0.0748	2.5148
Mar20_11th#3-2_5pm15	5	GAM42a	+	12	256	82	1.41	8.28	11.68	0.39	0.55	0.05	0.0131	0.0137	0.2554	0.1124	5.5370	0.6223	0.0132	0.2676
Mar20_11th#3-2_5pm15	6	GAM42a	-	12	256	182	2.05	9.96	20.41	0.47	0.96	0.14	0.0273	0.0322	2.0197	3.9739	15.3441	60.9764	1.2974	9.4613
Mar20_11th#3-2_5pm15	7	GAM42a	+	12	256	167	1.89	9.98	18.87	0.47	0.88	0.13	0.0232	0.0268	1.5146	1.4946	14.0229	20.9591	0.4459	3.5585
Mar20_12th#3-2_5pm20	1	GAM42a	+	12	256	375	2.94	11.73	34.49	0.55	1.62	0.34	0.0261	0.0305	1.8643	2.8093	38.0830	106.9872	2.2763	6.6886
Mar20_12th#3-2_5pm20	2	GAM42a	-	12	256	146	2.1	8.80	18.48	0.41	0.87	0.10	0.0225	0.0259	1.4289	1.3001	10.8974	14.1678	0.3014	3.0954
Mar20_13th#3-2_1pm30	1	GAM42a	+	24	427	245	2.34	10.74	25.12	1.01	1.41	0.86	0.0271	0.0319	1.9873	3.6761	95.8588	352.3902	7.4977	8.7523
Mar20_13th#3-2_1pm30	2	GAM42a	+	24	427	166	1.63	10.83	17.65	1.02	0.99	0.53	0.0276	0.0326	2.0566	4.3639	59.2241	258.4510	5.4990	10.3899
Mar20_13th#3-2_1pm30	3	GAM42a	-	24	427	238	1.95	11.71	22.84	1.10	1.28	0.87	0.0298	0.0355	2.3265	11.5490	97.2028	1122.5990	23.8851	27.4966
Mar20_13th#3-2_1pm30	4	GAM42a	-	24	427	426	1.78	16.50	29.36	1.55	1.65	2.13	0.0283	0.0335	2.1402	5.5200	238.5658	1316.8767	28.0187	13.1422
Mar20_13th#3-2_1pm30	5	GAM42a	-	24	427	249	1.44	14.25	20.53	1.34	1.15	0.99	0.0279	0.0329	2.0885	4.7525	111.1697	528.3338	11.2411	11.3150
Mar20_13th#3-2_1pm30	6	GAM42a	-	24	427	167	2.08	9.46	19.68	0.89	1.11	0.50	0.0277	0.0326	2.0605	4.4084	56.0469	247.0791	5.2570	10.4958
Mar20_13th#3-2_1pm30	7	GAM42a	-	24	427	341	5.99	7.68	46.03	0.72	2.59	0.96	0.0280	0.0330	2.0972	4.8694	107.0348	521.1920	11.0892	11.5932
Mar20_13th#3-2_1pm30	8	GAM42a	+	24	427	232	2.04	11.27	23.00	1.06	1.29	0.82	0.0258	0.0302	1.8341	2.6434	92.3163	244.0282	5.1921	6.2935
Mar20_13th#3-2_1pm30	9	GAM42a	+	24	427	257	2.71	10.15	27.50	0.95	1.55	0.87	0.0269	0.0317	1.9685	3.5187	97.7425	343.9254	7.3176	8.3775
Mar20_13th#3-2_1pm30	10	GAM42a	+	24	427	388	1.78	15.74	28.02	1.48	1.58	1.85	0.0250	0.0292	1.7374	2.1978	207.3678	455.7572	9.6970	5.2327
Mar20_13th#3-2_1pm30	11	GAM42a	+	24	427	235	2.37	10.44	24.75	0.98	1.39	0.80	0.0245	0.0285	1.6737	1.9593	89.6595	175.6723	3.7377	4.6649
Mar20_13th#3-2_1pm30	12	GAM42a	+	24	427	254	1.39	14.70	20.43	1.38	1.15	1.03	0.0260	0.0304	1.8522	2.7414	115.0245	315.3249	6.7090	6.5268
Mar20_13th#3-2_1pm30	13	GAM42a	+	24	427	225	1.67	12.43	20.76	1.17	1.17	0.83	0.0247	0.0288	1.6994	2.0511	92.9686	190.6902	4.0572	4.8834
Mar20_13th#3-2_1pm30	14	GAM42a	+	24	427	377	2.97	11.70	34.74	1.10	1.95	1.50	0.0263	0.0309	1.8936	2.9854	167.7338	500.7576	10.6544	7.1079
Mar20_13th#3-2_1pm30	15	GAM42a	+	24	427	382	4.9	9.03	44.24	0.85	2.49	1.24	0.0258	0.0301	1.8256	2.5994	138.8392	360.8951	7.6786	6.1887
Mar20_13th#3-2_1pm30	16	GAM42a	-	24	427	351	2.1	13.64	28.65	1.28	1.61	1.52	0.0274	0.0322	2.0218	3.9954	170.2755	680.3106	14.4747	9.5123
Mar20_13th#3-2_1pm30	17	GAM42a	+	24	427	368	2.57	12.50	32.12	1.17	1.81	1.53	0.0270	0.0317	1.9730	3.5558	170.7587	607.1781	12.9187	8.4658
Mar20_13th#3-2_1pm30	18	GAM42a	+	24	427	285	2.52	11.12	28.02	1.04	1.57	1.05	0.0266	0.0312	1.9236	3.1835	117.2028	373.1106	7.9385	7.5793
Mar20_13th#3-2_1pm30	19	GAM42a	+	24	427	463	2.29	14.94	34.20	1.40	1.92	2.24	0.0261	0.0306	1.8690	2.8368	250.8516	711.6118	15.1407	6.7540
Mar20_13th#3-2_1pm30	20	GAM42a	+	24	427	234	1.8	12.15	21.87	1.14	1.23	0.87	0.0266	0.0312	1.9281	3.2146	96.8470	311.3211	6.6239	7.6534
Mar20_13th#3-2_1pm30	21	GAM42a	+	24	427	303	1.88	13.49	25.36	1.26	1.43	1.26	0.0253	0.0296	1.7725	2.3466	141.0666	331.0231	7.0430	5.5868
Mar20_13th#3-2_1pm30	22	GAM42a	+	24	427	184	2.14	9.78	20.92	0.92	1.18	0.57	0.0270	0.0318	1.9817	3.6284	64.2466	233.1124	4.9598	8.6387
Mar20_13th#3-2_1pm30	23	GAM42a	+	24	427	183	2.13	9.77	20.82	0.92	1.17	0.57	0.0252	0.0294	1.7601	2.2925	63.8178	146.3016	3.1128	5.4581
Mar20_13th#3-2_1pm30	24	GAM42a	+	24	427	224	2.43	10.06	24.43	0.94	1.37	0.74	0.0259	0.0303	1.8425	2.6883	82.7203	222.3758	4.7314	6.4004
Mar20_13th#3-2_1pm30	25	GAM42a	+	24	427	246	2.23	11.05	24.64	1.04	1.38	0.88	0.0257	0.0301	1.8215	2.5784	98.0089	252.7100	5.3768	6.1389
Mar20_13th#3-2_1pm30	26	GAM42a	+	24	427	354	1.88	14.58	27.41	1.37	1.54	1.59	0.0241	0.0280	1.6201	1.7846	178.1415	317.9084	6.7640	4.2488
Mar20_13th#3-2_1pm30	27	GAM42a	-	24	427	181	2.34	9.23	21.59	0.87	1.21	0.54	0.0249	0.0290	1.7164	2.1149	60.8696	128.7346	2.7390	5.0353
Mar20_13th#3-2_1pm30	28	GAM42a	-	24	427	265	2.2	11.55	25.42	1.08	1.43	0.98	0.0239	0.0278	1.6027	1.7323	110.0646	190.6664	4.0567	4.1244
Mar20_13th#3-2_1pm30	29	GAM42a	-	24	427	322	2.33	12.34	28.75	1.16	1.62	1.29	0.0290	0.0344	2.2246	7.3351	144.6431	1060.9733	22.5739	17.4638
Mar20_13th#3-2_1pm30	30	GAM42a	+	24	427	164	1.59	10.92	17.36	1.02	0.98	0.52	0.0253	0.0296	1.7751	2.3579	58.4513	137.8230	2.9324	5.6138
Mar20_13th#3-2_1pm30	31	GAM42a	-	24	427	193	3.32	7.88	26.17	0.74	1.47	0.53	0.0249	0.0290	1.7209	2.1325	58.7957	125.3815	2.6677	5.0772
Mar20_13th#3-2_1pm30	32	GAM42a	-	24	427	155	3.11	7.32	22.75	0.69	1.28	0.39	0.0234	0.0271	1.5359	1.5484	43.4314	67.2475	1.4308	3.6864
