# Supplementary material for: Isotopic tracing reveals single-cell assimilation of a macroalgal polysaccharide by a few marine Flavobacteria and Gammaproteobacteria
Source: ISME J. 2021 May 5;15(10):3062–75. doi: 10.1038/s41396-021-00987-x (PMC8443679; doi:10.1038/s41396-021-00987-x)
Supplement: Supplementary file 4 — Table S3 [file 41396_2021_987_MOESM4_ESM.pdf]

Table S3: Functional gene annotation of fosmid inserts

CDS #	Length	Annotation			Best blastp hit					
	(bp)	Function	Gene	EC number	Organism	Order	% ID	eval	Description	Accession
Fosmid F10 insert										
1	594	Alginate lyase precursor		4.2.2.3	*Psychromonas* sp. RZ22	*Alteromonadales*	80.2	4.00E-107	polysaccharide lyase family 7	WP_134277012.1
2	1611	Alginate lyase precursor		4.2.2.3	*Catenovulum agarivorans* DS2	*Alteromonadales*	81.5	0	hypothetical protein	EWH10908.1
3	654	Sodium-type flagellar protein MotX			*Psychromonas* sp. SP041	*Alteromonadales*	73.37	6.00E-97	sel1 repeat family protein	WP_025565356.1
4	765	Phosphoadenylyl-sulfate reductase [thioredoxin]	cysH	1.8.4.8	*Psychromonas* sp. RZ5	*Alteromonadales*	84.25	8.00E-164	Phosphoadenylyl-sulfate reductase	WP_134291061.1
5	1752	Sulfite reductase [NADPH] hemoprotein beta-component	cysI	1.8.1.2	*Psychromonas* sp. RZ5	*Alteromonadales*	91.77	0.00E+00	assimilatory sulfite reductase (NADPH) hemoprotein subunit	WP_134291060.1
6	1803	Sulfite reductase [NADPH] flavoprotein alpha-component	cysJ	1.8.1.2	*Psychromonas* sp. RZ5	*Alteromonadales*	88.35	0.00E+00	assimilatory sulfite reductase (NADPH) flavoprotein subunit	WP_134291059.1
7	1338	hypothetical protein			*Psychromonas hadalis*	*Alteromonadales*	69.8	0.00E+00	MipA/OmpV family protein	WP_022942365.1
8	627	hypothetical protein			*Psychromonas arctica*	*Alteromonadales*	68.42	1.00E-93	TIGR04219 family outer membrane beta-barrel protein	WP_028869192.1
9	435	Copper metallochaperone PCu(A)C, inserts Cu(I) into cytochrome oxidase subunit II			*Psychromonas* sp. RZ22	*Alteromonadales*	65.89	4.00E-60	copper chaperone PCu(A)C	WP_134276360.1
10	210	hypothetical protein			*Psychromonas* sp. RZ5	*Alteromonadales*	85.29	2.00E-29	hypothetical protein	WP_134291056.1
11	501	Acetolactate synthase small subunit	ilvH	2.2.1.6	*Psychromonas* sp. RZ22	*Alteromonadales*	87.95	1.00E-100	Acetolactate synthase small subunit	WP_134276358.1
12	1725	Acetolactate synthase large subunit	ilvI	2.2.1.6	*Psychromonas ossibalaenae*	*Alteromonadales*	89.2	0.00E+00	Acetolactate synthase 3 large subunit	WP_019613644.1
13	432	Thioredoxin 2	trxC	1.8.1.8	*Halarcobacter mediterraneus*	*Campylobacterales*	41.04	2.00E-29	thioredoxin TrxC	WP_129060805.1
14	1050	Peptide chain release factor 2 @ programmed frameshift-containing	prfB		*Psychromonas* sp. RZ22	*Alteromonadales*	90.72	0.00E+00	Peptide chain release factor	WP_134276356.1
15	1518	Lysyl-tRNA synthetase (class II)	lysS	6.1.1.6	*Psychromonas* sp. SA13A	*Alteromonadales*	95.25	0.00E+00	lysine-tRNA ligase	WP_166372516.1
16	480	hypothetical protein			*Psychromonas* sp. SR45-3	*Alteromonadales*	61.59	5.00E-41	hypothetical protein	WP_182690171.1
17	1482	Sensory histidine kinase BaeS	baeS	2.7.13.3	*Psychromonas* sp. L1A2	*Alteromonadales*	59.72	0.00E+00	HAMP domain-containing protein	WP_160063805.1
18	684	Response regulator BaeR	baeR		*Psychromonas aquimarina*	*Alteromonadales*	77.58	4.00E-122	response regulator	WP_028863610.1
19	453	LSU ribosomal protein L9p	rplI		*Psychromonas aquimarina*	*Alteromonadales*	92.09	3.00E-77	50S ribosomal protein L9	WP_028865158.1
20	228	SSU ribosomal protein S18p @ SSU ribosomal protein S18p, zinc-independent	rpsR		*Psychromonas* sp. psych-6C06	*Alteromonadales*	98.67	6.00E-47	30S ribosomal protein S18	WP_101107902.1
21	291	Primosomal replication protein N	priB		*Psychromonas* sp. RZ5	*Alteromonadales*	86.46	4.00E-55	Primosomal replication protein N	WP_134291044.1
22	396	SSU ribosomal protein S6p	rpsF		*Psychromonas arctica*	*Alteromonadales*	95	1.00E-79	30S ribosomal protein S6	WP_028869179.1
23	1368	hypothetical protein			*Psychromonas* sp. RZ22	*Alteromonadales*	50.32	2.00E-149	DUF481 domain-containing protein	WP_134276348.1
24	744	23S rRNA (guanosine(2251)-2'-O)-methyltransferase	rlmB	2.1.1.185	*Psychromonas* sp. B3M02	*Alteromonadales*	90.61	1.00E-163	23S rRNA (guanosine(2251)-2'-O)-methyltransferase RlmB	WP_113820521.1
25	2493	exoribonuclease RNase R	rnr	3.1.13.1	*Psychromonas* sp. RZ22	*Alteromonadales*	84.23	0.00E+00	ribonuclease R	WP_134276346.1
26	927	hypothetical protein			*Psychromonas* sp. RZ5	*Alteromonadales*	72.08	3.00E-166	tripartite tricarboxylate transporter substrate binding protein	WP_134291290.1
27	495	hypothetical protein			*Psychromonas* sp. B3M02	*Alteromonadales*	49.68	4.00E-45	hypothetical protein	WP_113818330.1
28	924	Porin-like protein H	ompH		*Psychromonas* sp. L1A2	*Alteromonadales*	49.84	2.00E-88	porin	WP_160063793.1
29	1323	hypothetical protein			*Psychromonas* sp. RZ22	*Alteromonadales*	70.45	0.00E+00	BamA/TamA family outer membrane protein	WP_134276332.1
30	417	hypothetical protein			*Psychromonas* sp. Urea-02u-13	*Alteromonadales*	49.64	3.00E-38	hypothetical protein	WP_101081879.1
31	528	Molybdopterin-guanine dinucleotide biosynthesis protein MobB	mobB		*Psychromonas* sp. SP041	*Alteromonadales*	64.16	2.00E-76	Molybdopterin-guanine dinucleotide biosynthesis protein MobB	WP_025566056.1
32	510	hypothetical protein			*Psychromonas* sp. RZ22	*Alteromonadales*	48.24	2.00E-46	hypothetical protein	WP_134276329.1
33	732	Uncharacterized conserved protein YfiP, contains DTW domain			*Psychromonas* sp. RZ22	*Alteromonadales*	75	1.00E-130	DTW domain-containing protein	WP_134276328.1
Fosmid F25 insert										
1	246	hypothetical protein			-	-	-	-	-	-
2	774	hypothetical protein			*Planctomyces* bacterium	*Planctomycetales*	41.8	3.00E-54	hypothetical protein	HBP21570.1
3	819	Nitric oxide -responding transcriptional regulator Dnr (Crp/Fnr family)			*Colwellia psychrerythraea*	*Alteromonadales*	62.6	7.00E-104	type IV pili methyl-accepting chemotaxis transducer N-terminal d	WP_033093395.1
4	1734	Putative lyase			*Psychromonas* sp. RZ5	*Alteromonadales*	70.92	8.00E-122	polysaccharide lyase	WP_134291789.1
5	1521	Competence protein ComM	comM		*Colwellia agarivorans*	*Alteromonadales*	81.3	0.00E+00	YifB family Mg chelatase-like AAA ATPase	WP_077339884.1
6	1662	Acetolactate synthase isozyme 2 large subunit	ilvG	2.2.1.6	*Colwellia agarivorans*	*Alteromonadales*	83.33	0.00E+00	acetolactate synthase 2 catalytic subunit	WP_077339882.1
7	246	Acetolactate synthase isozyme 2 small subunit	ilvM	2.2.1.6	*Colwellia agarivorans*	*Alteromonadales*	84.42	8.00E-37	acetolactate synthase 2 small subunit	WP_077339880.1
8	1857	Dihydroxy-acid dehydratase	ilvD	4.2.1.9	*Colwellia* sp. UCD-KL20	*Alteromonadales*	95.63	0.00E+00	dihydroxy-acid dehydratase	WP_076417502.1
9	1569	L-threonine dehydratase biosynthetic IlvA	ilvA	4.3.1.19	*Colwellia* sp. PAMC 21821	*Alteromonadales*	76.97	0.00E+00	threonine ammonia-lyase, biosynthetic	WP_155866729.1
10	1338	Sensor histidine kinase RcsC	rcsC	2.7.13.3	*Thalassotalea profundi*	*Alteromonadales*	64.99	0.00E+00	response regulator	WP_189377855.1
11	264	Ubiquinone biosynthesis accessory factor UbiK	ubiK		*Thalassotalea* sp. HSM 43	*Alteromonadales*	79.49	8.00E-35	accessory factor UbiK family protein	WP_135438569.1
12	2019	ATP-dependent DNA helicase Rep	rep	3.6.4.12	*Colwellia* sp. UCD-KL20	*Alteromonadales*	89.7	0.00E+00	DNA helicase Rep	WP_076417497.1
13	657	hypothetical protein			*Cellvibrio zantedeschiae*	*Cellvibrionales*	57.45	4.00E-65	hypothetical protein	GGY78368.1
14	1914	Methyl-accepting chemotaxis protein PctC	pctC_1		*Catenovulum sediminis*	*Alteromonadales*	56.6	0.00E+00	HAMP domain-containing protein	WP_185976773.1
15	1914	Methyl-accepting chemotaxis protein PctC	pctC_2		*Catenovulum sediminis*	*Alteromonadales*	55.73	0.00E+00	HAMP domain-containing protein	WP_185976773.1
16	2244	Exo-oligoalginate lyase		4.2.2.26	*Colwellia echini*	*Alteromonadales*	78.06	0.00E+00	alginate lyase family protein	WP_101345554.1
17	1104	hypothetical protein			*Colwellia echini*	*Alteromonadales*	57.34	7.00E-135	GGDEF domain-containing protein	WP_101345192.1
18	885	Hydrogen peroxide-inducible genes activator	oxyR		*Colwellia* sp. 20A7	*Alteromonadales*	73.72	5.00E-159	LysR family transcriptional regulator	WP_159817135.1
19	387	hypothetical protein			-	-	-	-	-	-
20	2052	Vitamin B12 transporter BtuB	btuB		*Paraglaciecola* sp. L3A3	*Alteromonadales*	60.5	0.00E+00	TonB-dependent receptor	WP_158971995.1
21	1104	hypothetical protein			*Catenovulum* sp. CCB-QB4	*Alteromonadales*	50.94	2.00E-113	HmuY family protein	WP_108601873.1
22	660	hypothetical protein			*Catenovulum* sp. RQJ05	*Alteromonadales*	79.78	5.00E-95	MotA/TolQ/ExbB proton channel family protein	WP_111977473.1
23	306	hypothetical protein			*Colwellia echini*	*Alteromonadales*	97.03	1.00E-62	DUF2149 domain-containing protein	WP_101342650.1
24	4263	Aerobic cobaltochelatase subunit CobN	cobN	6.6.1.2	*Colwellia echini*	*Alteromonadales*	78.1	0.00E+00	cobaltochelatase subunit CobN	WP_101342651.1
25	2310	Photosystem I assembly protein Ycf3	ycf3		*Vibrio vulnificus*	*Vibrionales*	51.5	0.00E+00	hypothetical protein	WP_157401374.1
26	969	hypothetical protein			*Catenovulum* sp. CCB-QB4	*Alteromonadales*	49.32	8.00E-91	alpha/beta hydrolase	WP_108602152.1
