# Supplementary figures and images for: Isotopic tracing reveals single-cell assimilation of a macroalgal polysaccharide by a few marine Flavobacteria and Gammaproteobacteria
Source: ISME J. 2021 May 5;15(10):3062–75. doi: 10.1038/s41396-021-00987-x (PMC8443679; doi:10.1038/s41396-021-00987-x)

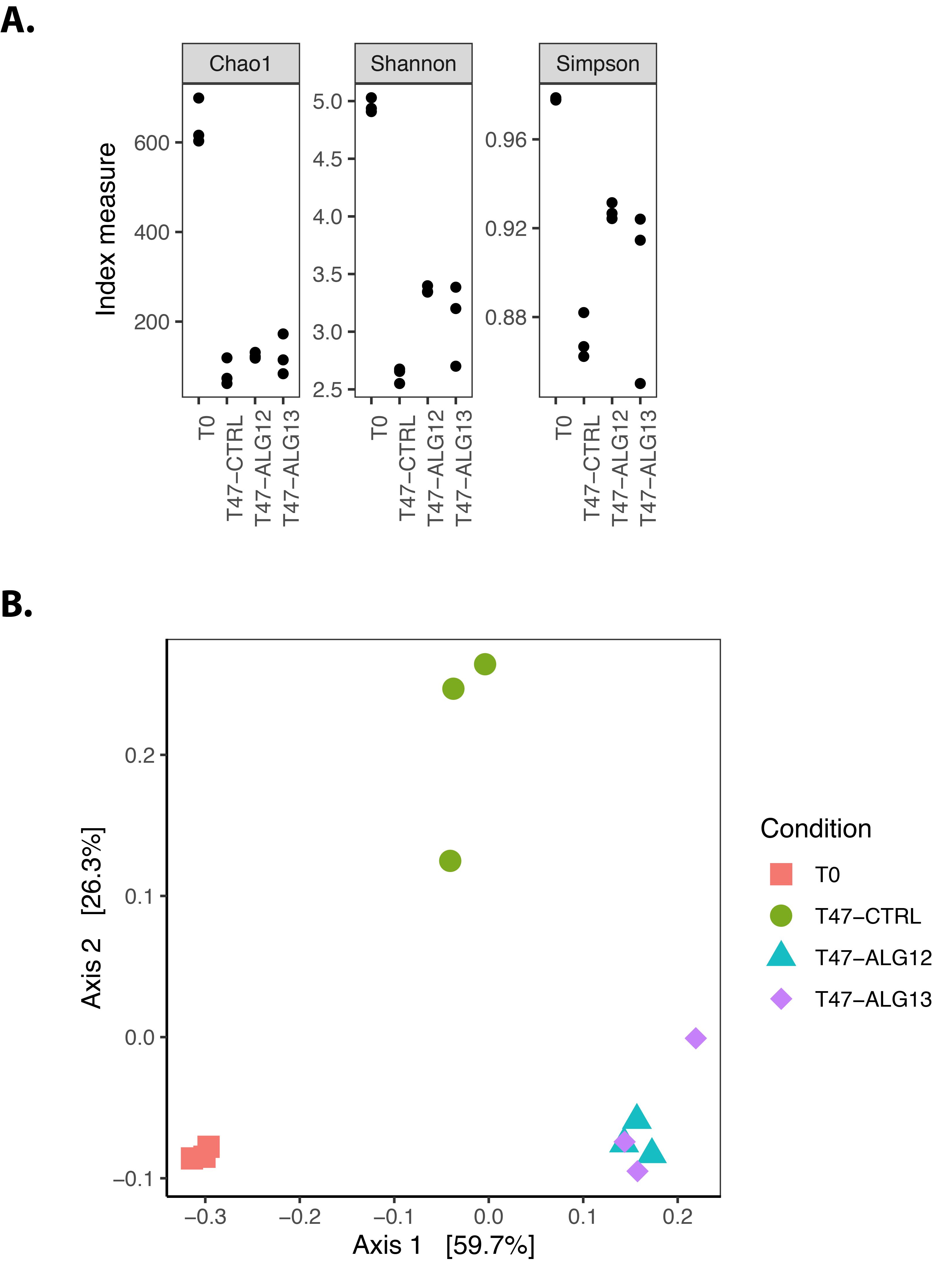

Supplement: Supplementary file 5 — Figure S1 [file 41396_2021_987_MOESM5_ESM.png]

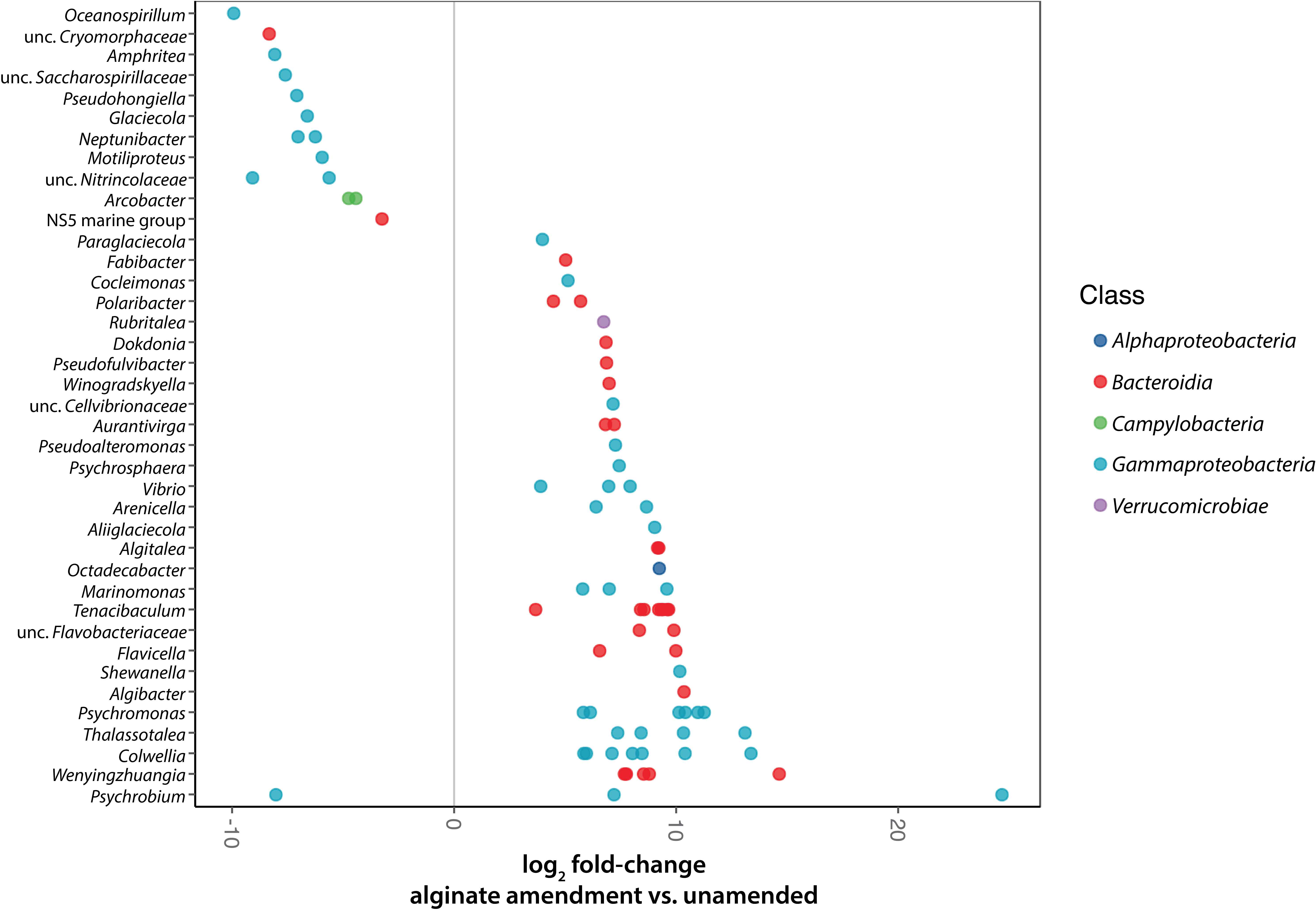

Supplement: Supplementary file 6 — Figure S2 [file 41396_2021_987_MOESM6_ESM.png]

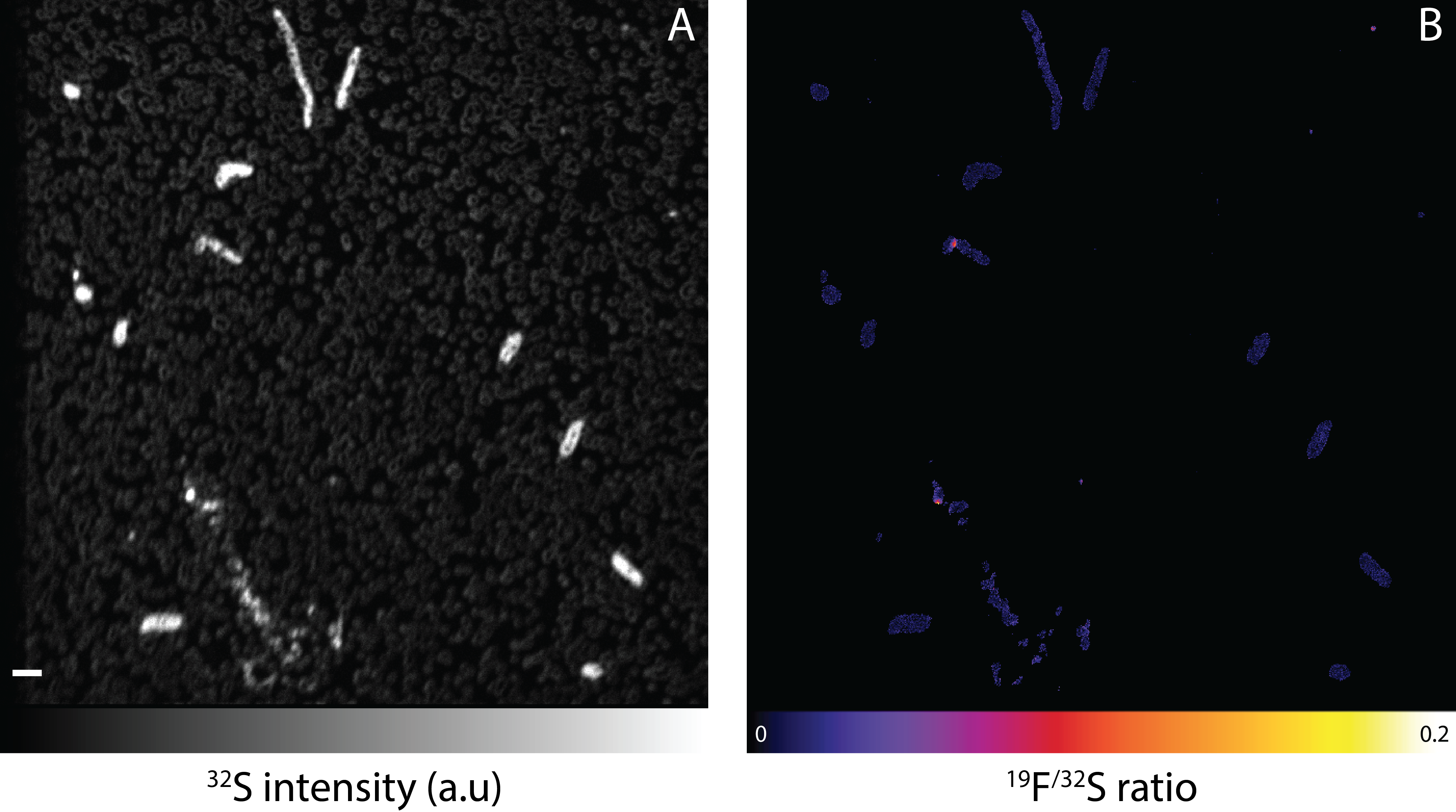

Supplement: Supplementary file 7 — Figure S3 [file 41396_2021_987_MOESM7_ESM.png]

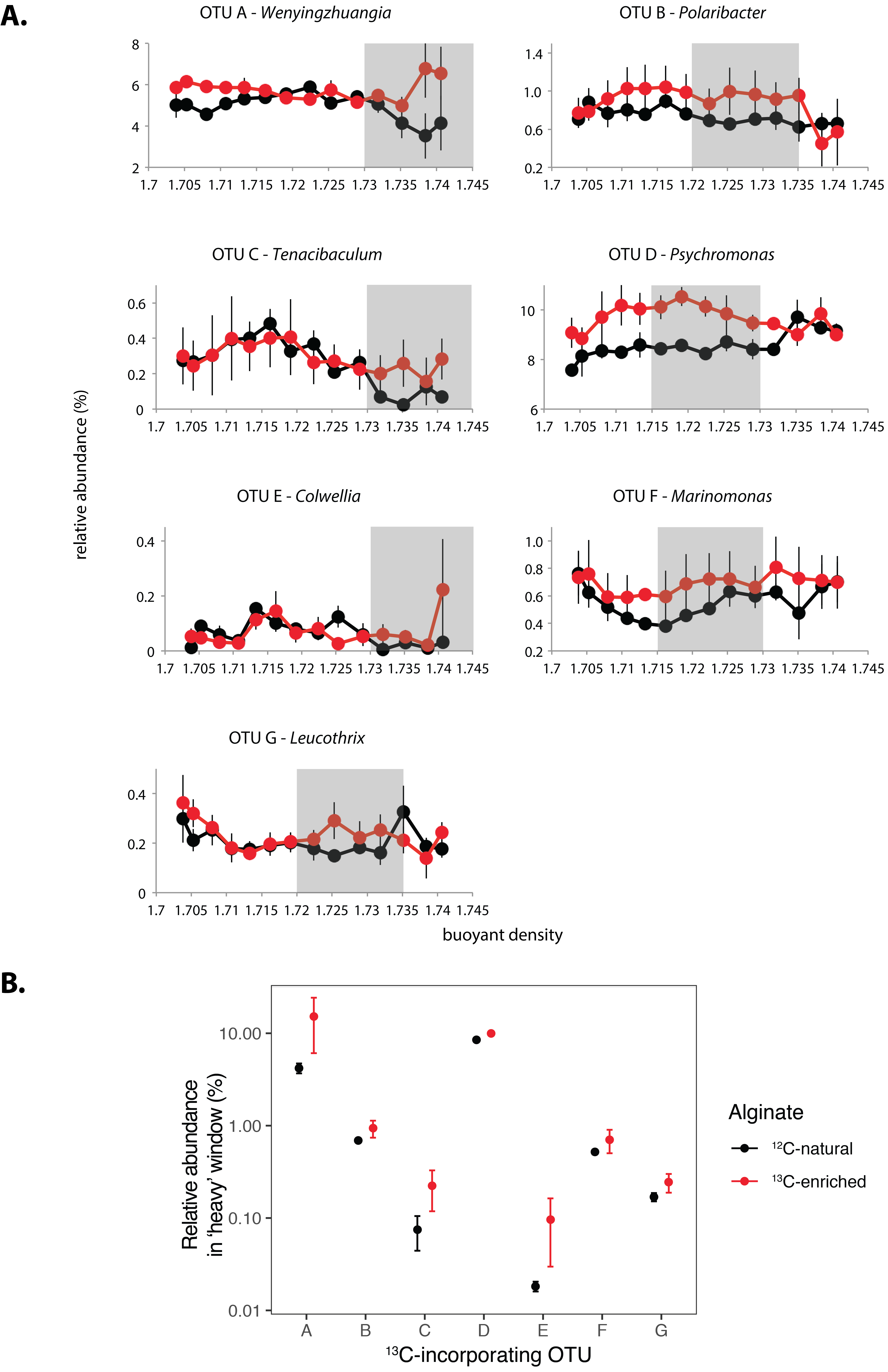

Supplement: Supplementary file 8 — Figure S4 [file 41396_2021_987_MOESM8_ESM.png]
